# Supplementary figures and images for: Rare variants contribute disproportionately to quantitative trait variation in yeast
Source: eLife. 2019 Oct 24;8:e49212. doi: 10.7554/eLife.49212 (PMC6892613; doi:10.7554/eLife.49212)

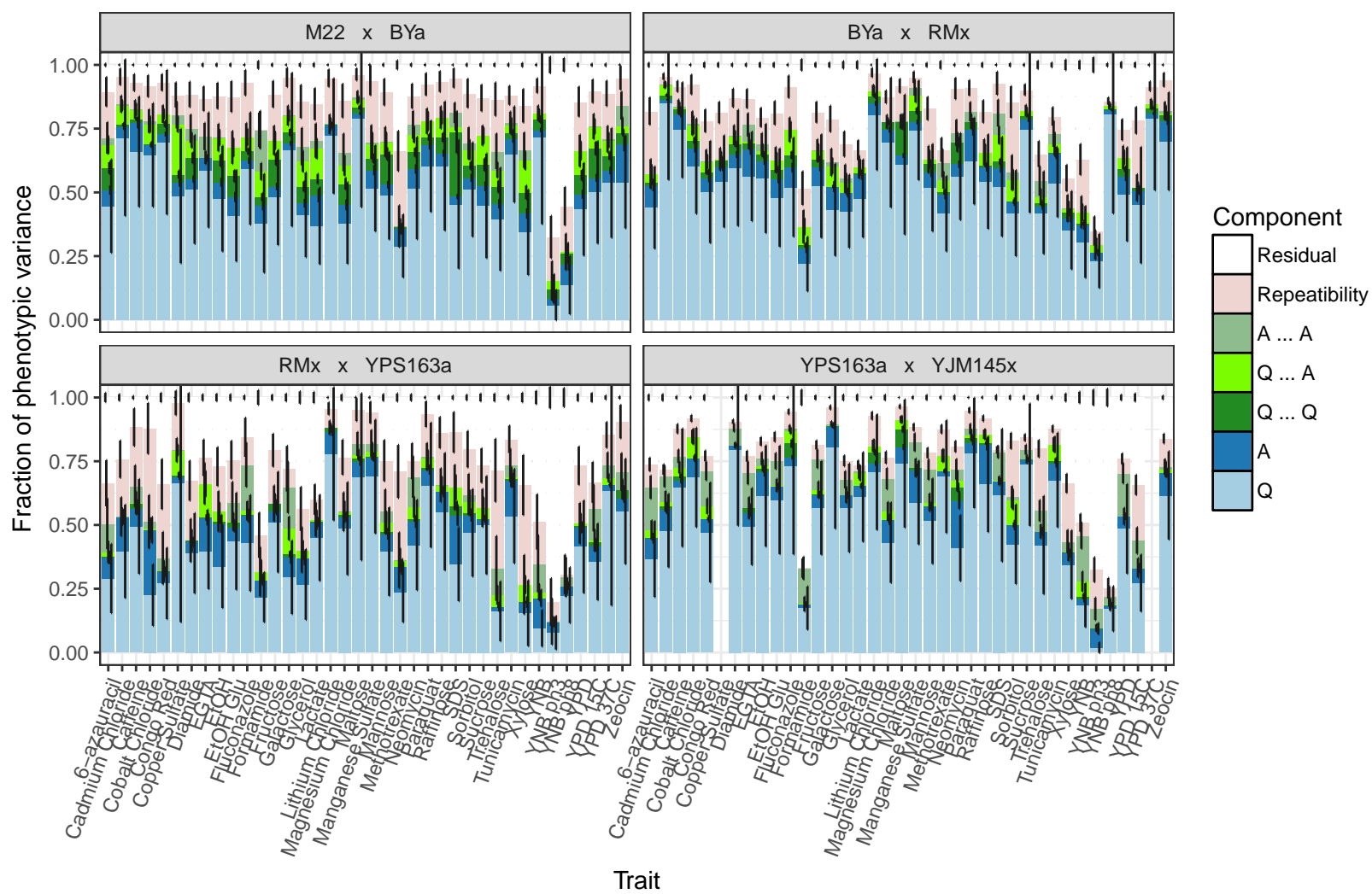

Fraction of phenotypic variance

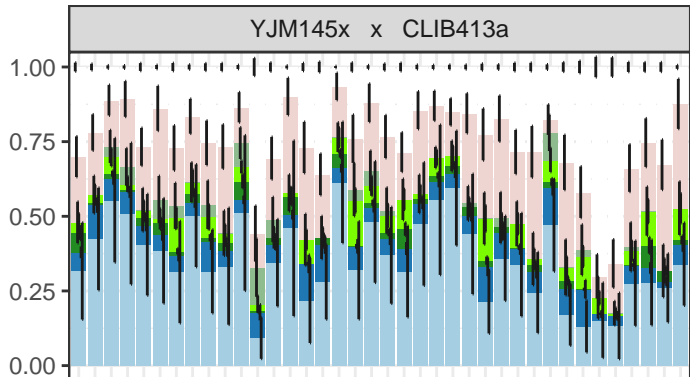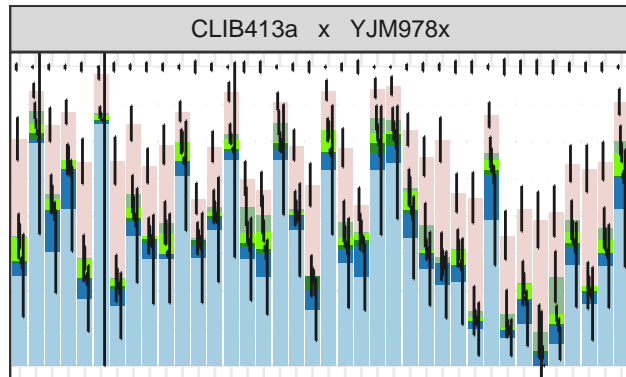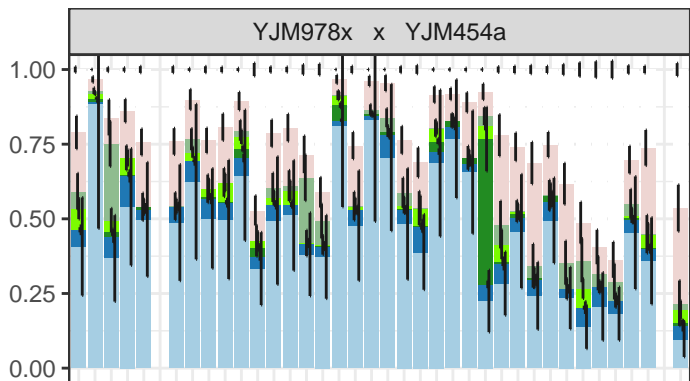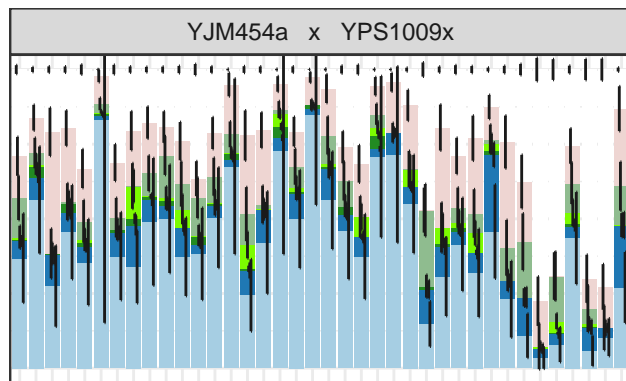

Component

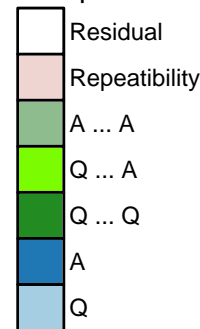

Trait

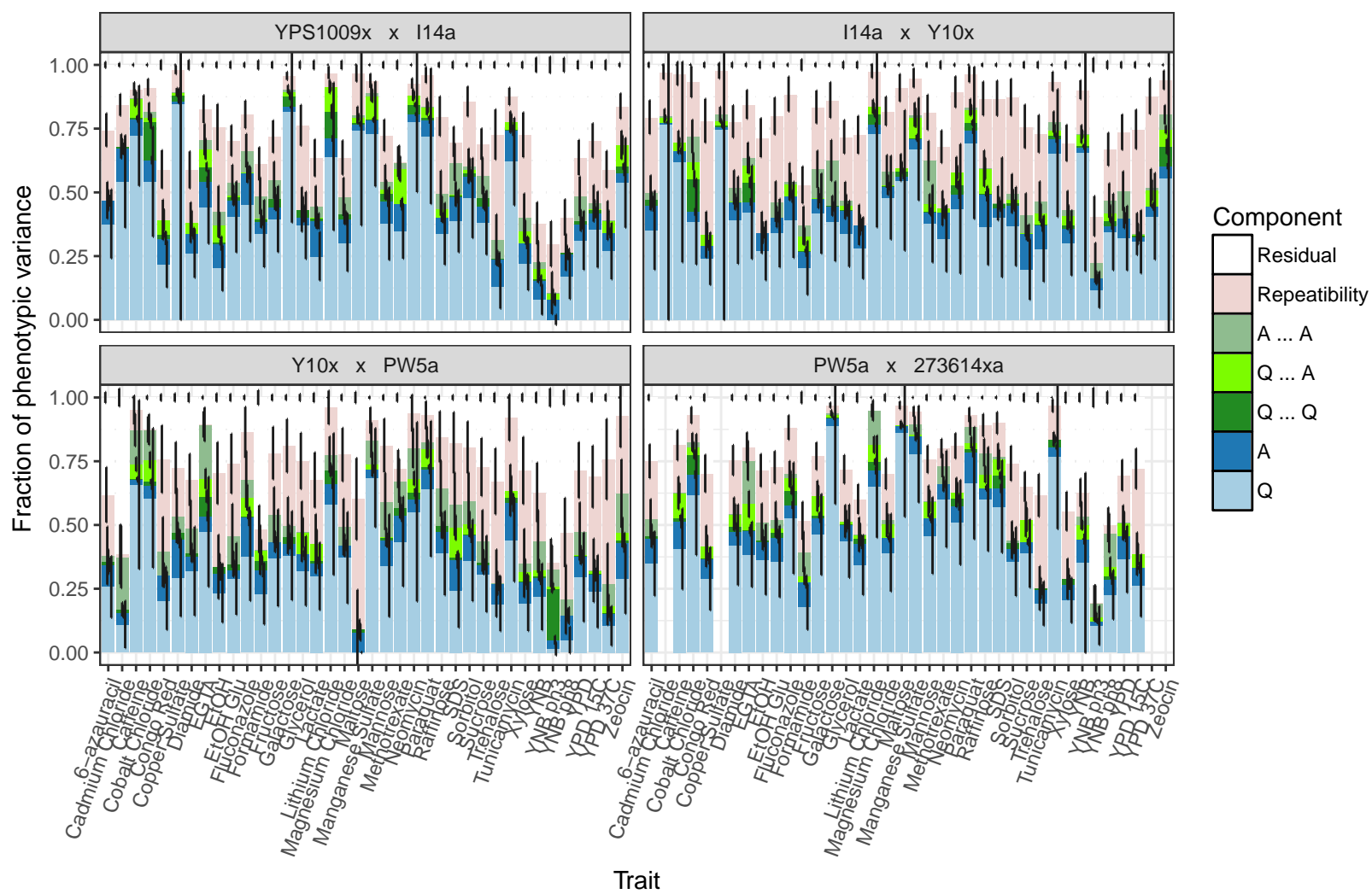

Trait

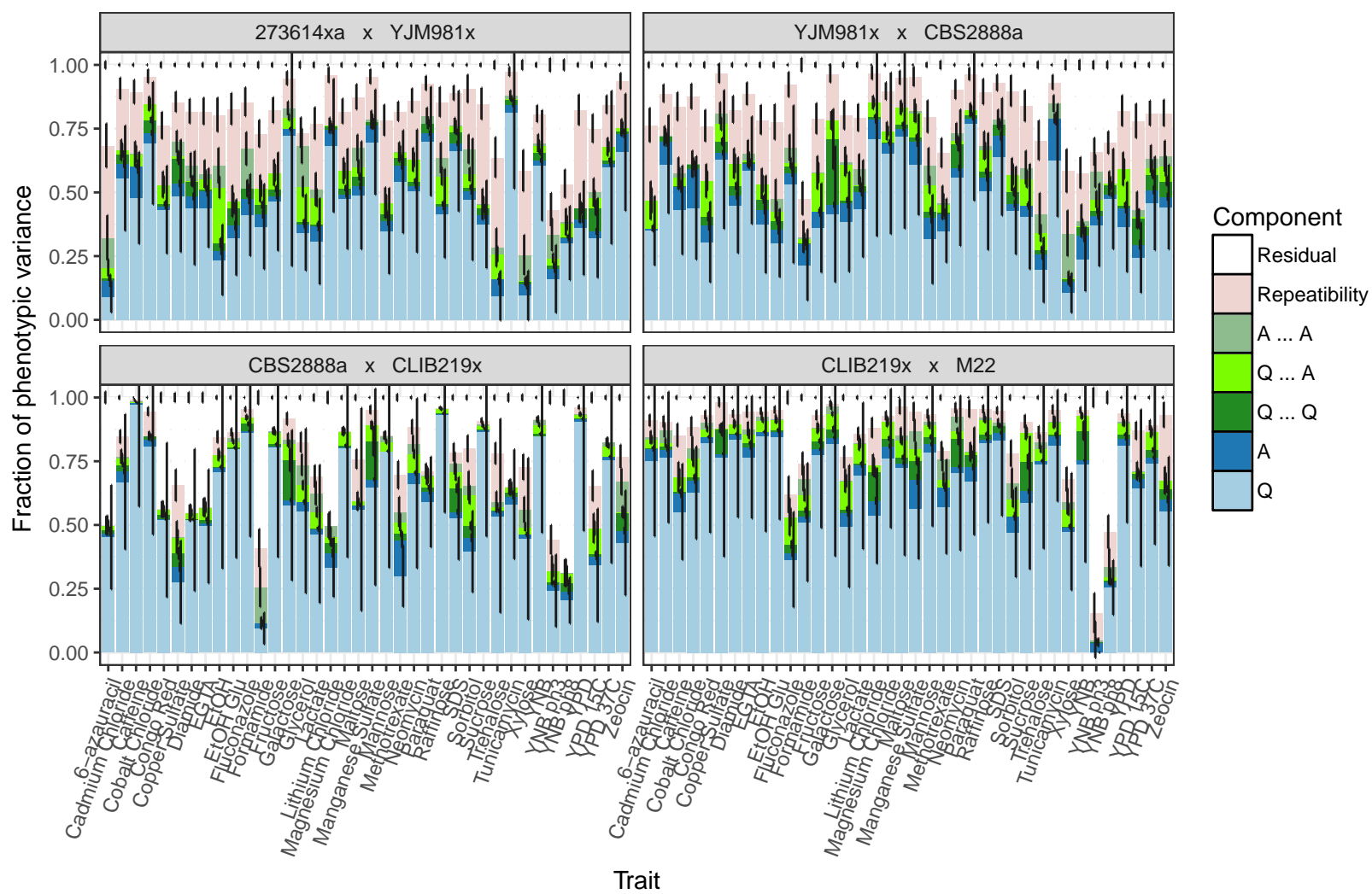

Supplement: Supplementary file 2. — For each trait and cross, a stacked barplot is shown representing the results from a multiple variance component model. Phenotypic covariance was modeled as the sum of QTL effects (light blue, denoted as Q), additive genome effects (dark blue, denoted as A), interactions between additive QTLs (dark green, denoted as Q∘Q), interactions between additive QTLs and the rest of the genome (light green, denoted as Q∘A), interactions between all loci in the genome (sea green, denoted as A∘A), residual effect of strain (pink), and residual error (white). [file elife-49212-supp2.pdf]
