# Supplementary material for: Rare variants contribute disproportionately to quantitative trait variation in yeast
Source: eLife. 2019 Oct 24;8:e49212. doi: 10.7554/eLife.49212 (PMC6892613; doi:10.7554/eLife.49212)
Supplement: Supplementary file 3. — Results from QTL mapping are shown for each trait. Each subpanel represents the results for a cross. Along the Y-axis the two parental strains for each cross are shown. Position of QTL along the genome is represented on the X-axis. The arrows represent QTL effects from the within-cross mapping. The arrows point toward the strain that increases growth. The size of the arrow reflects the magnitude of the QTL effect. Full length arrows represent QTL that explain more than 25% of phenotypic variance, ¾ length arrows represent QTL that explain between 8% and 25% of phenotypic variance, ½ length arrows represent QTL that explain between 4% and 8% of phenotypic variance, and short arrows represent QTL that explain less than 4% of phenotypic variance. Large effect QTL (explaining more than 4% of phenotypic variance) are colored black, and small effect QTL (less than 4% of phenotypic variance) are colored gray. The green vertical lines correspond to QTL detected from the joint QTL mapping analysis (Materials and methods). [file elife-49212-supp3.pdf]

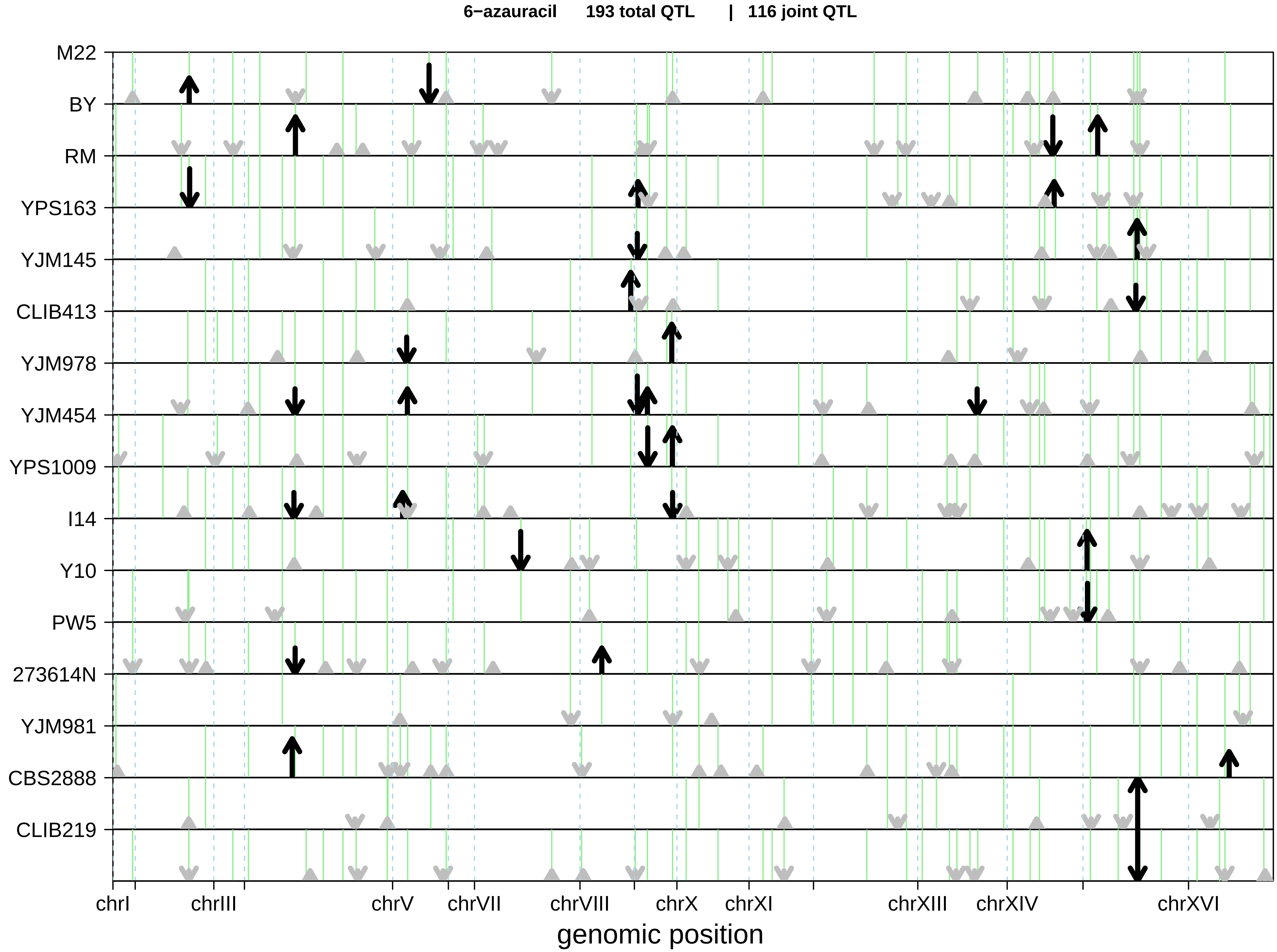

6−azauracil 193 total QTL | 116 joint QTL
M22
BY
RM
YPS163
YJM145
CLIB413
YJM978
YJM454
YPS1009
I14
Y10
PW5
273614N
YJM981
CBS2888
CLIB219
chrI
chrIII
chrV
chrVII
chrVIII
chrX
chrXI
chrXIII
chrXIV
chrXVI
genomic position

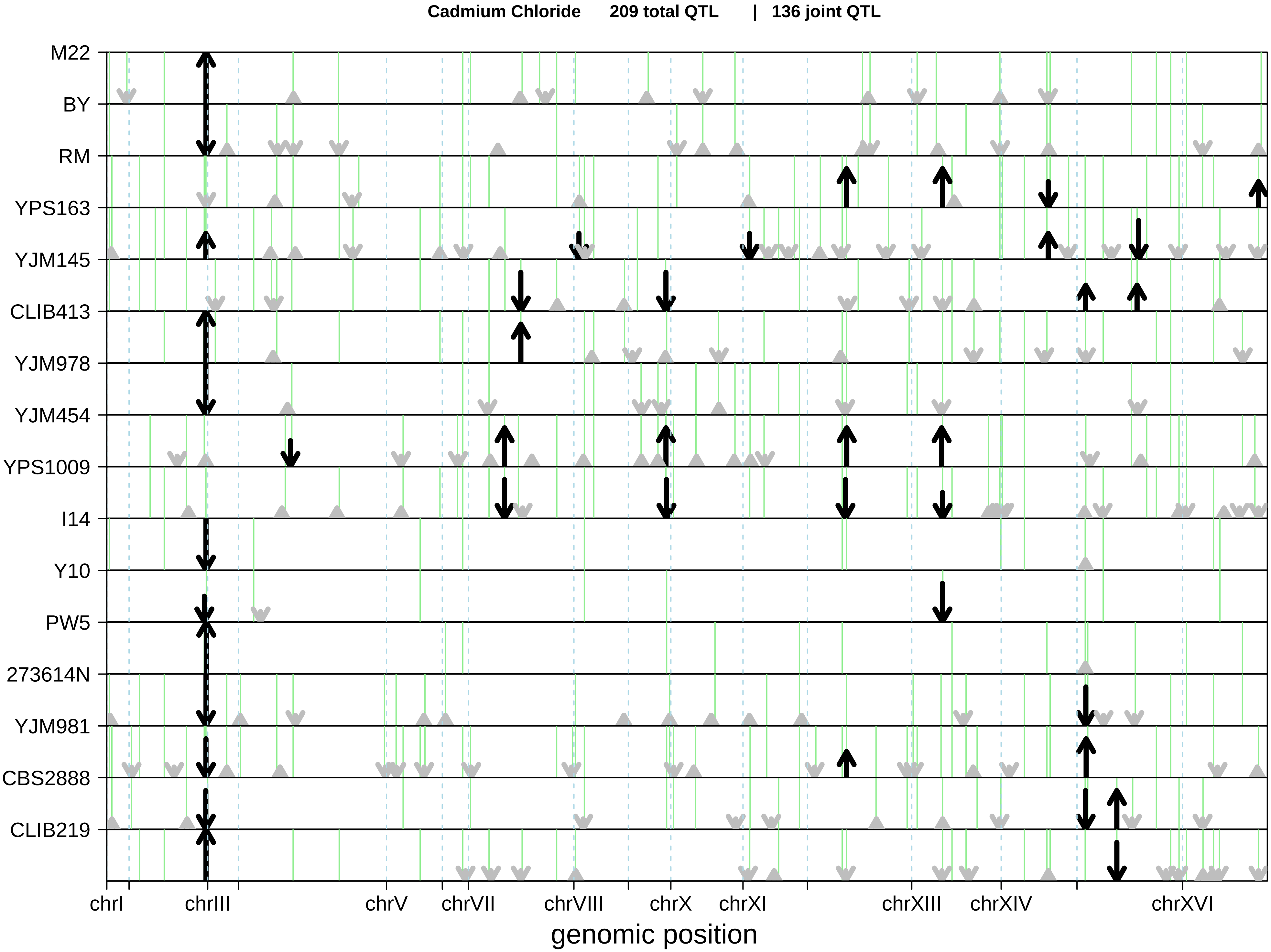

Cadmium Chloride 209 total QTL | 136 joint QTL
M22
BY
RM
YPS163
YJM145
CLIB413
YJM978
YJM454
YPS1009
I14
Y10
PW5
273614N
YJM981
CBS2888
CLIB219
chrI
chrIII
chrV
chrVII
chrVIII
chrX
chrXI
chrXIII
chrXIV
chrXVI
genomic position

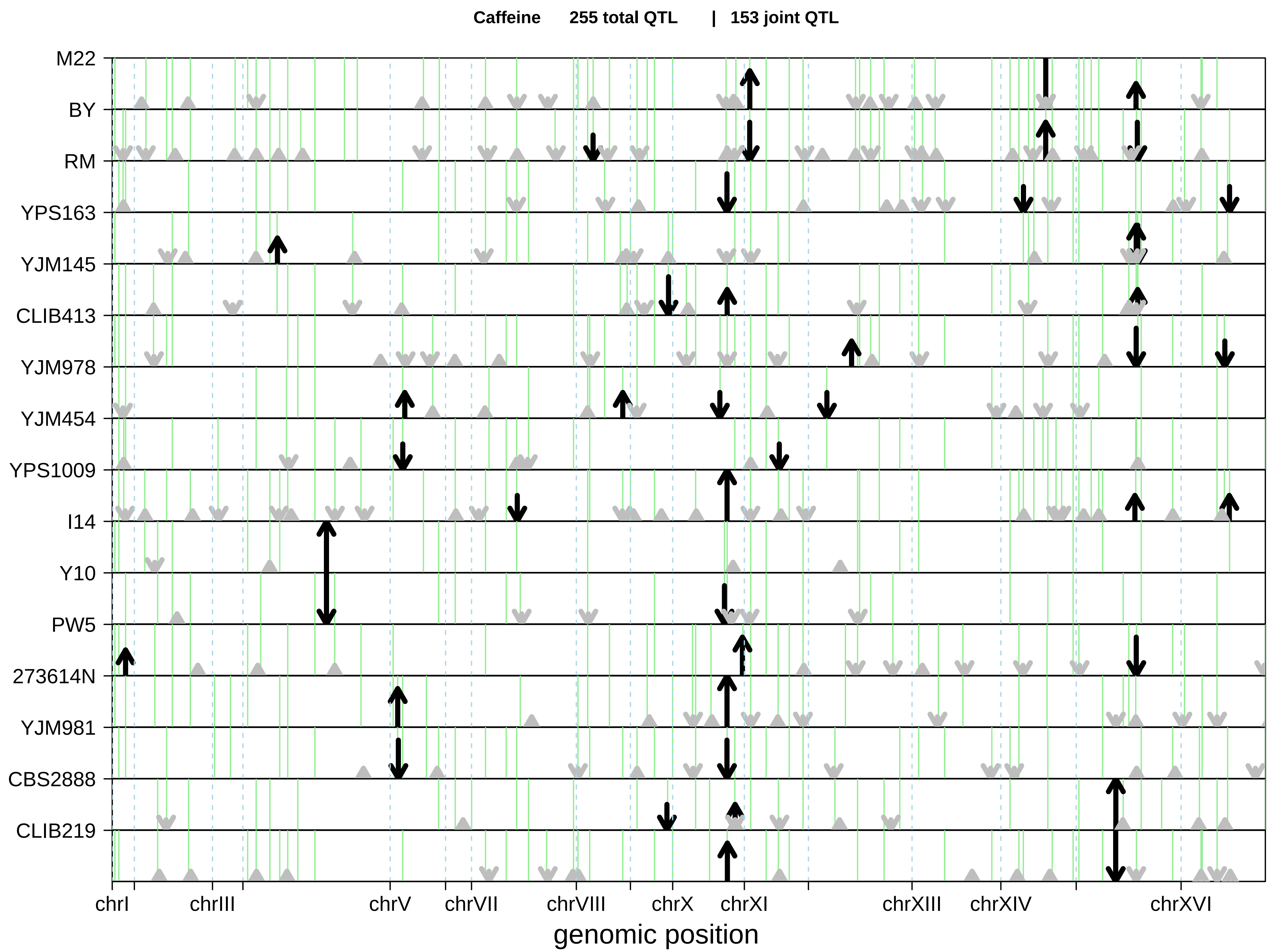
Caffeine 255 total QTL | 153 joint QTL
M22
BY
RM
YPS163
YJM145
CLIB413
YJM978
YJM454
YPS1009
I14
Y10
PW5
273614N
YJM981
CBS2888
CLIB219
chrI
chrIII
chrV
chrVII
chrVIII
chrX
chrXI
chrXIII
chrXIV
chrXVI
genomic position

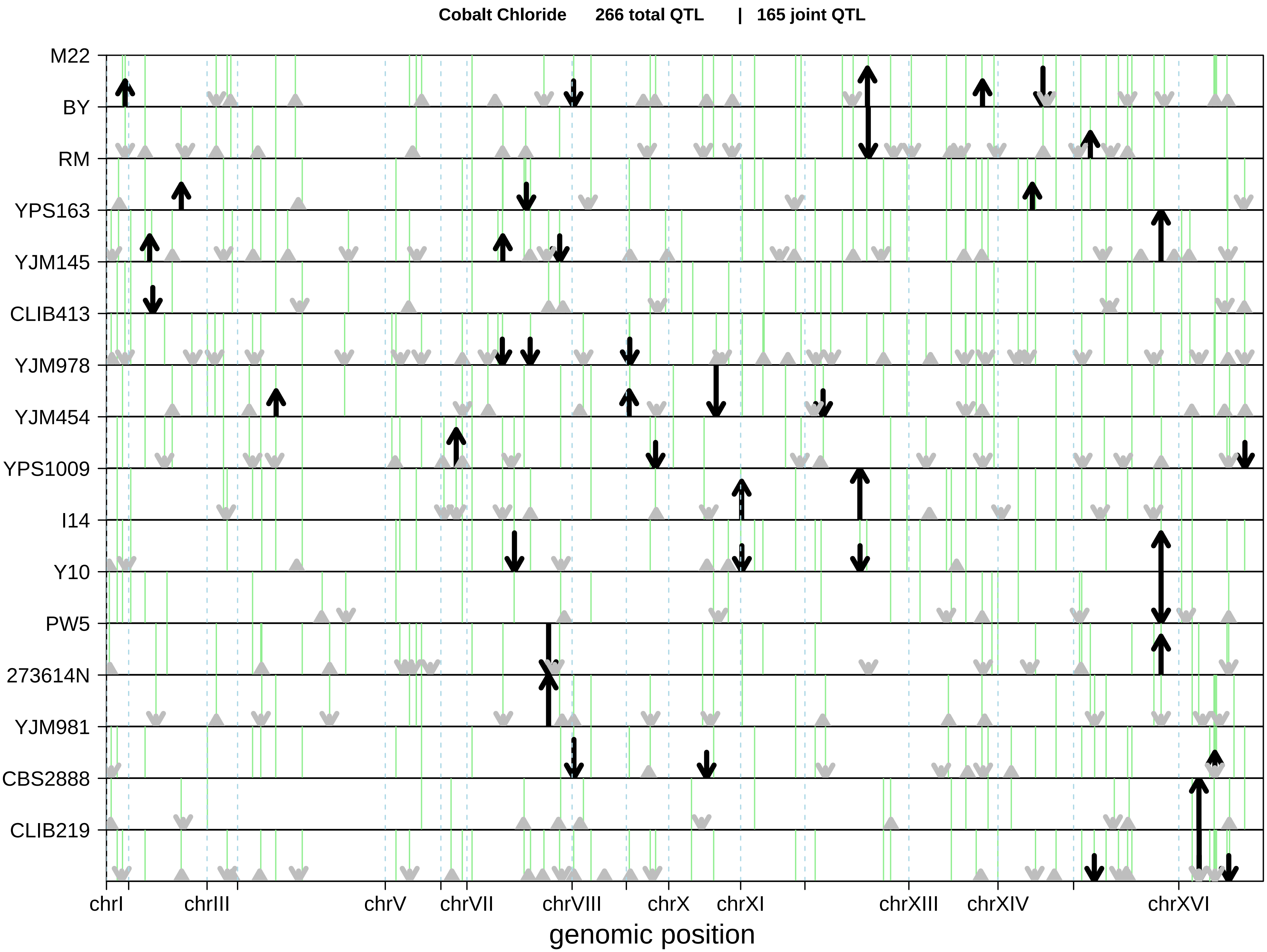

Cobalt Chloride 266 total QTL | 165 joint QTL
M22
BY
RM
YPS163
YJM145
CLIB413
YJM978
YJM454
YPS1009
I14
Y10
PW5
273614N
YJM981
CBS2888
CLIB219
chrI
chrIII
chrV
chrVII
chrVIII
chrX
chrXI
chrXIII
chrXIV
chrXVI
genomic position

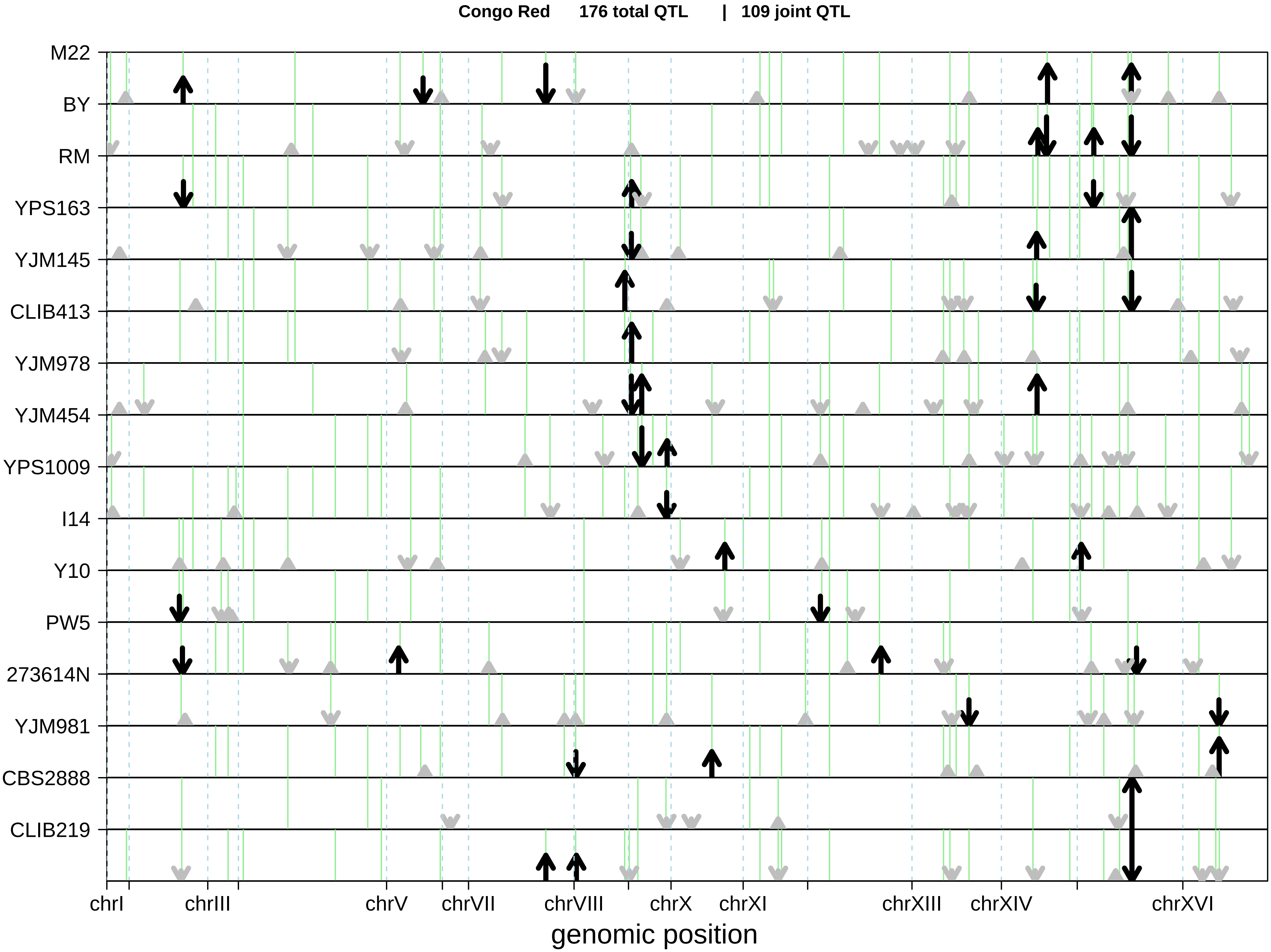
Congo Red 176 total QTL | 109 joint QTL
M22
BY
RM
YPS163
YJM145
CLIB413
YJM978
YJM454
YPS1009
I14
Y10
PW5
273614N
YJM981
CBS2888
CLIB219
chrI
chrIII
chrV
chrVII
chrVIII
chrX
chrXI
chrXIII
chrXIV
chrXVI
genomic position

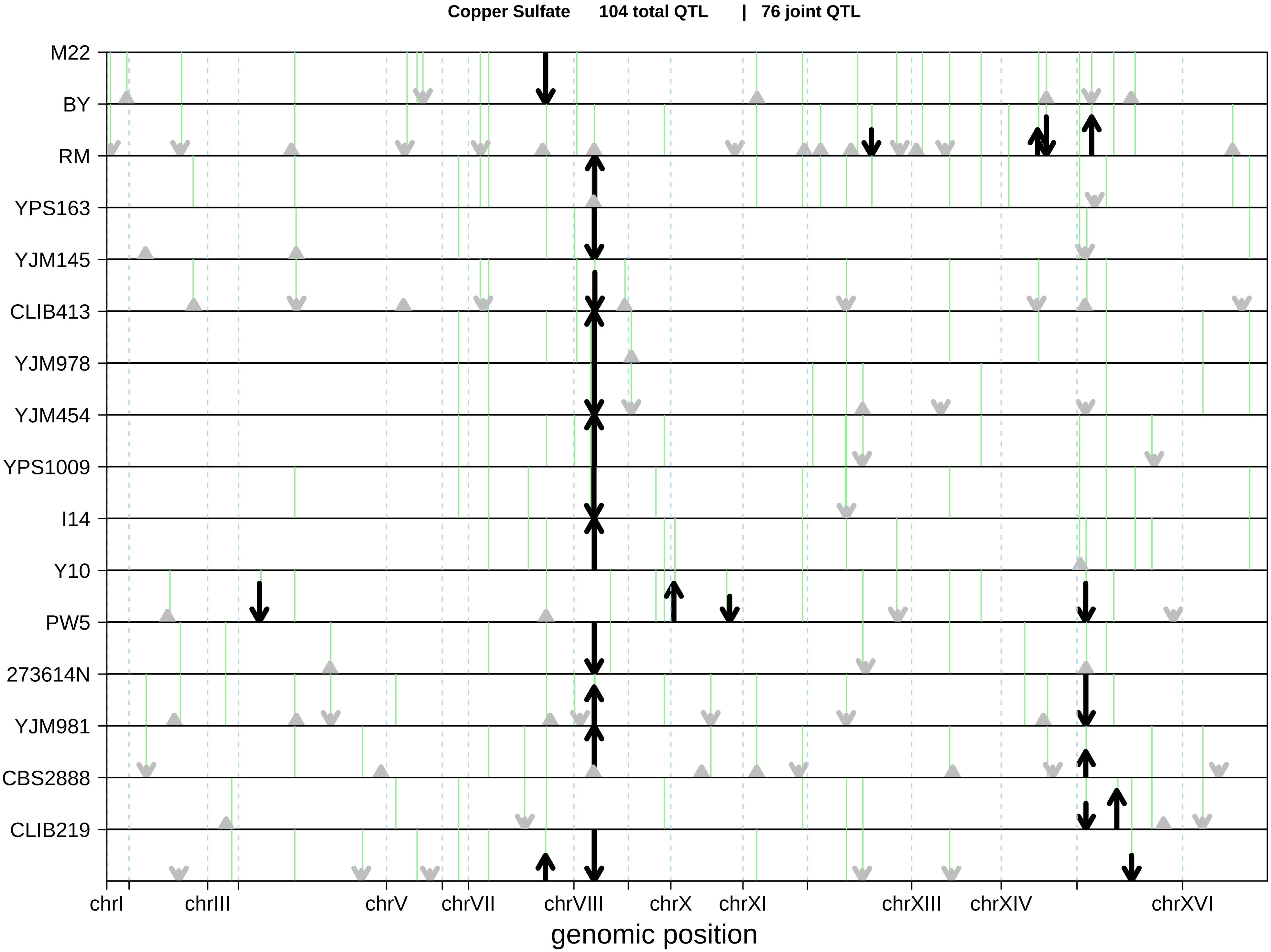
Copper Sulfate 104 total QTL | 76 joint QTL
M22
BY
RM
YPS163
YJM145
CLIB413
YJM978
YJM454
YPS1009
I14
Y10
PW5
273614N
YJM981
CBS2888
CLIB219
chrI
chrIII
chrV
chrVII
chrVIII
chrX
chrXI
chrXIII
chrXIV
chrXVI
genomic position

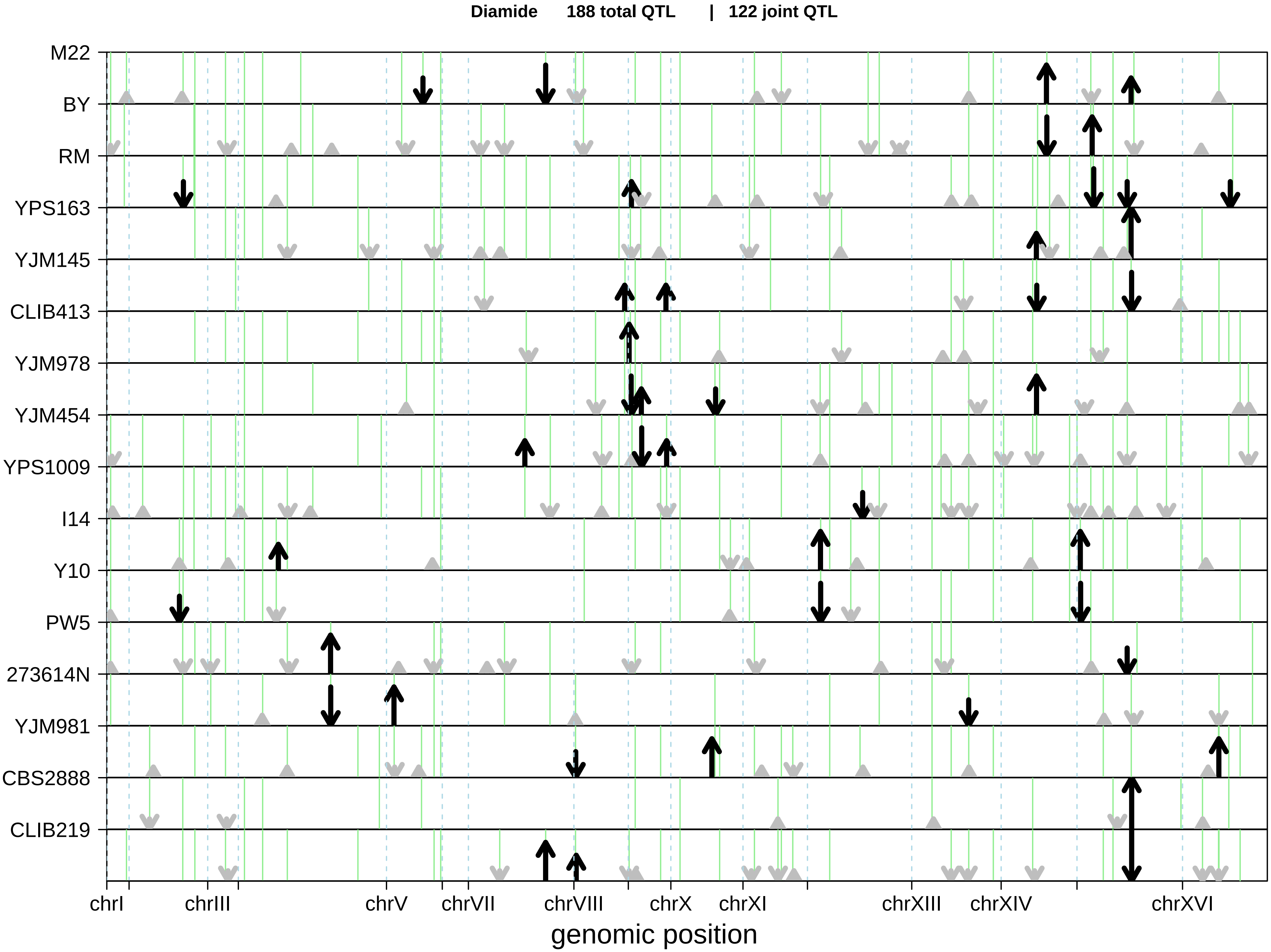

Diamide 188 total QTL | 122 joint QTL
M22
BY
RM
YPS163
YJM145
CLIB413
YJM978
YJM454
YPS1009
I14
Y10
PW5
273614N
YJM981
CBS2888
CLIB219
chrI
chrIII
chrV
chrVII
chrVIII
chrX
chrXI
chrXIII
chrXIV
chrXVI
genomic position

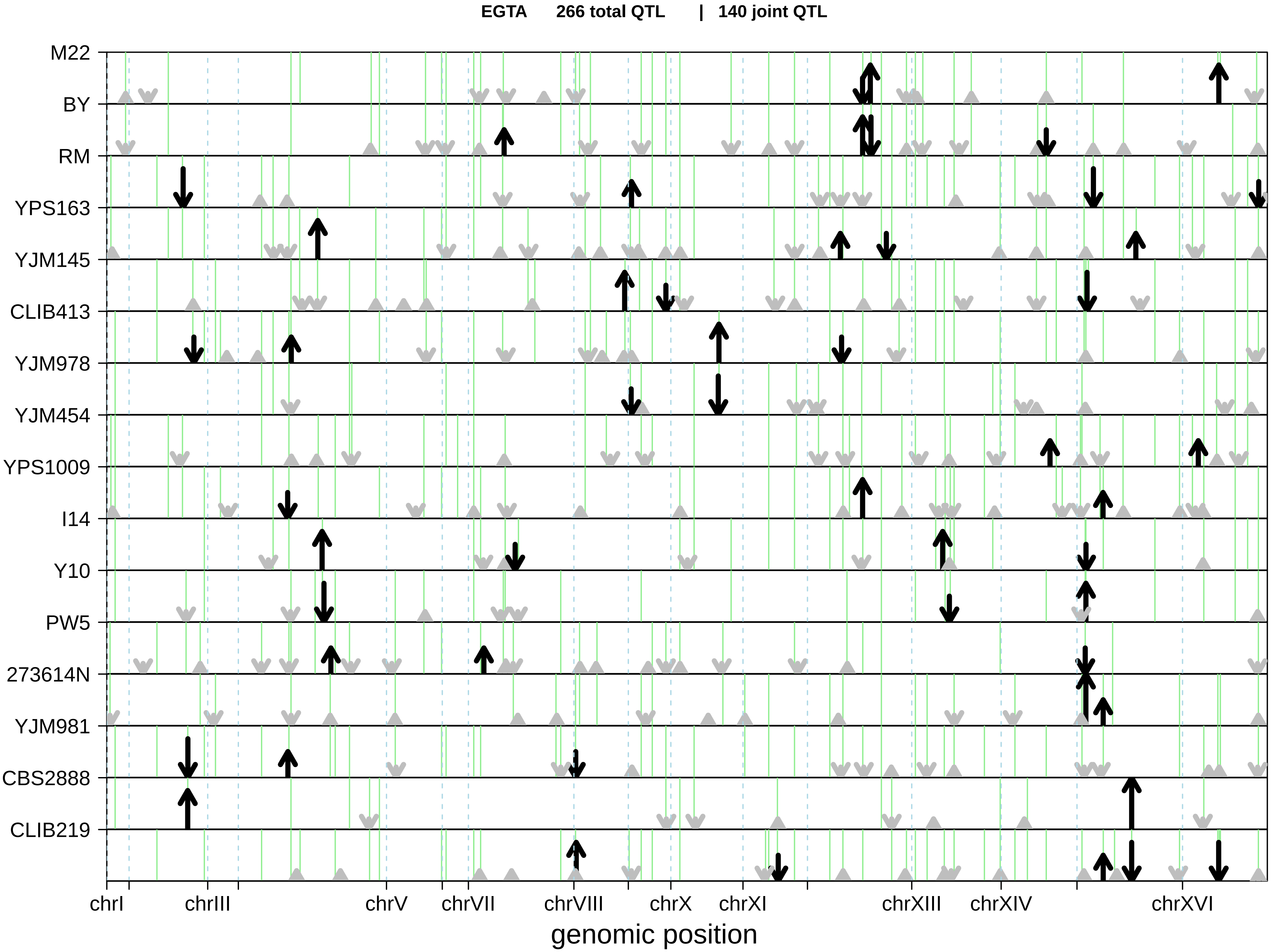

EGTA 266 total QTL | 140 joint QTL
M22
BY
RM
YPS163
YJM145
CLIB413
YJM978
YJM454
YPS1009
I14
Y10
PW5
273614N
YJM981
CBS2888
CLIB219
chrI
chrIII
chrV
chrVII
chrVIII
chrX
chrXI
chrXIII
chrXIV
chrXVI
genomic position

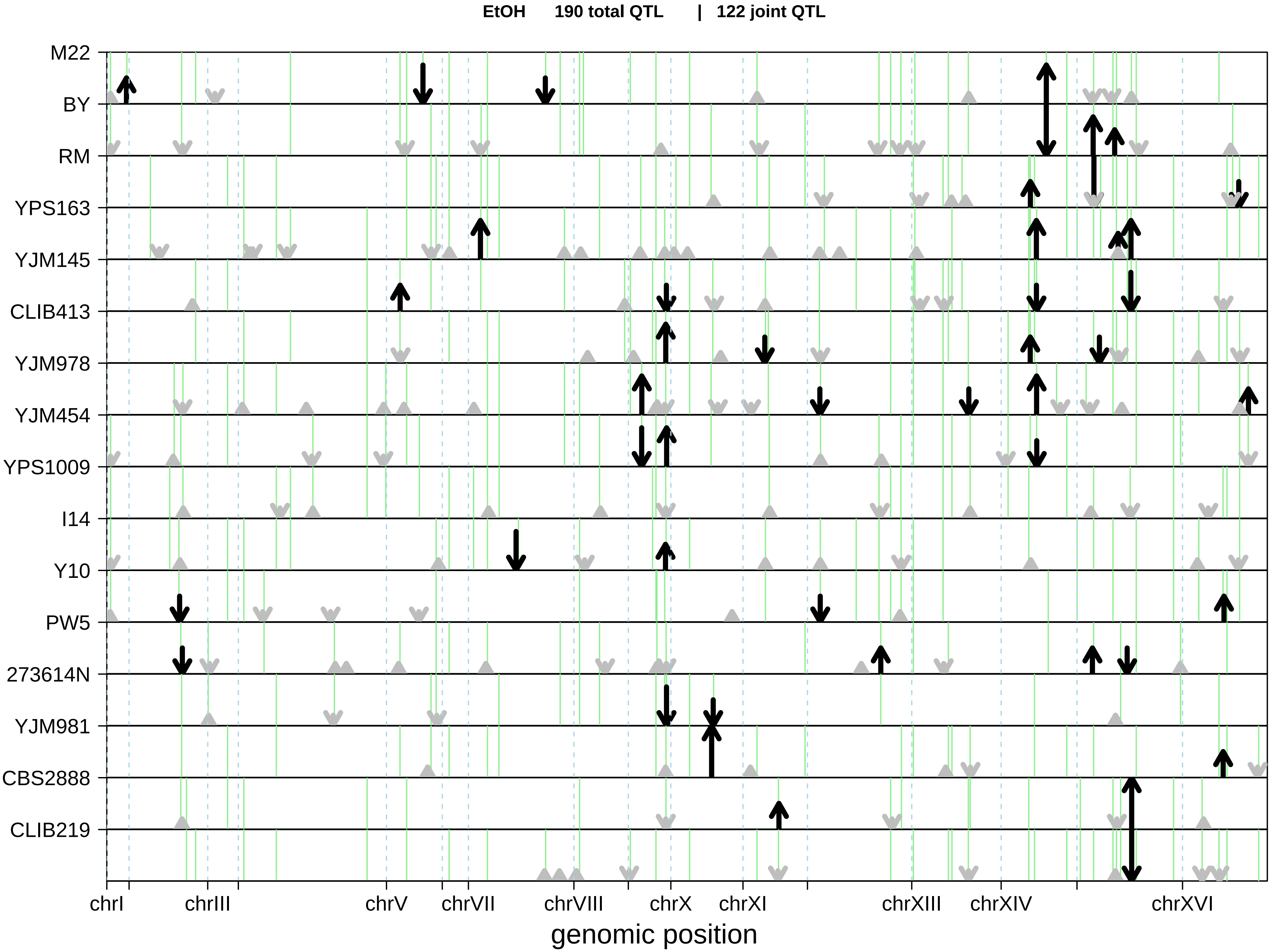
EtOH 190 total QTL | 122 joint QTL
M22
BY
RM
YPS163
YJM145
CLIB413
YJM978
YJM454
YPS1009
I14
Y10
PW5
273614N
YJM981
CBS2888
CLIB219
chrI
chrIII
chrV
chrVII
chrVIII
chrX
chrXI
chrXIII
chrXIV
chrXVI
genomic position

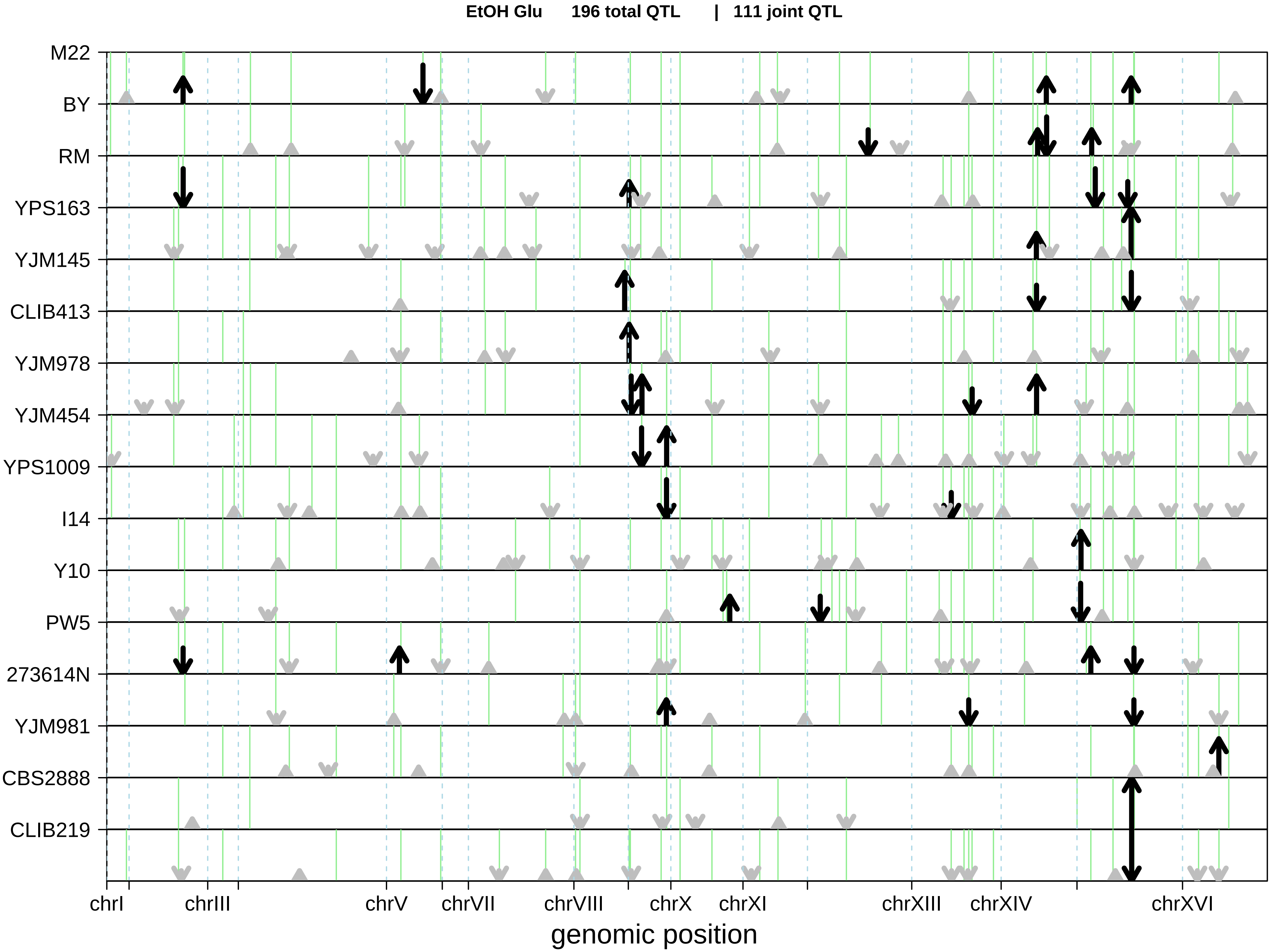
EtOH Glu 196 total QTL | 111 joint QTL
M22
BY
RM
YPS163
YJM145
CLIB413
YJM978
YJM454
YPS1009
I14
Y10
PW5
273614N
YJM981
CBS2888
CLIB219
chrI
chrIII
chrV
chrVII
chrVIII
chrX
chrXI
chrXIII
chrXIV
chrXVI
genomic position

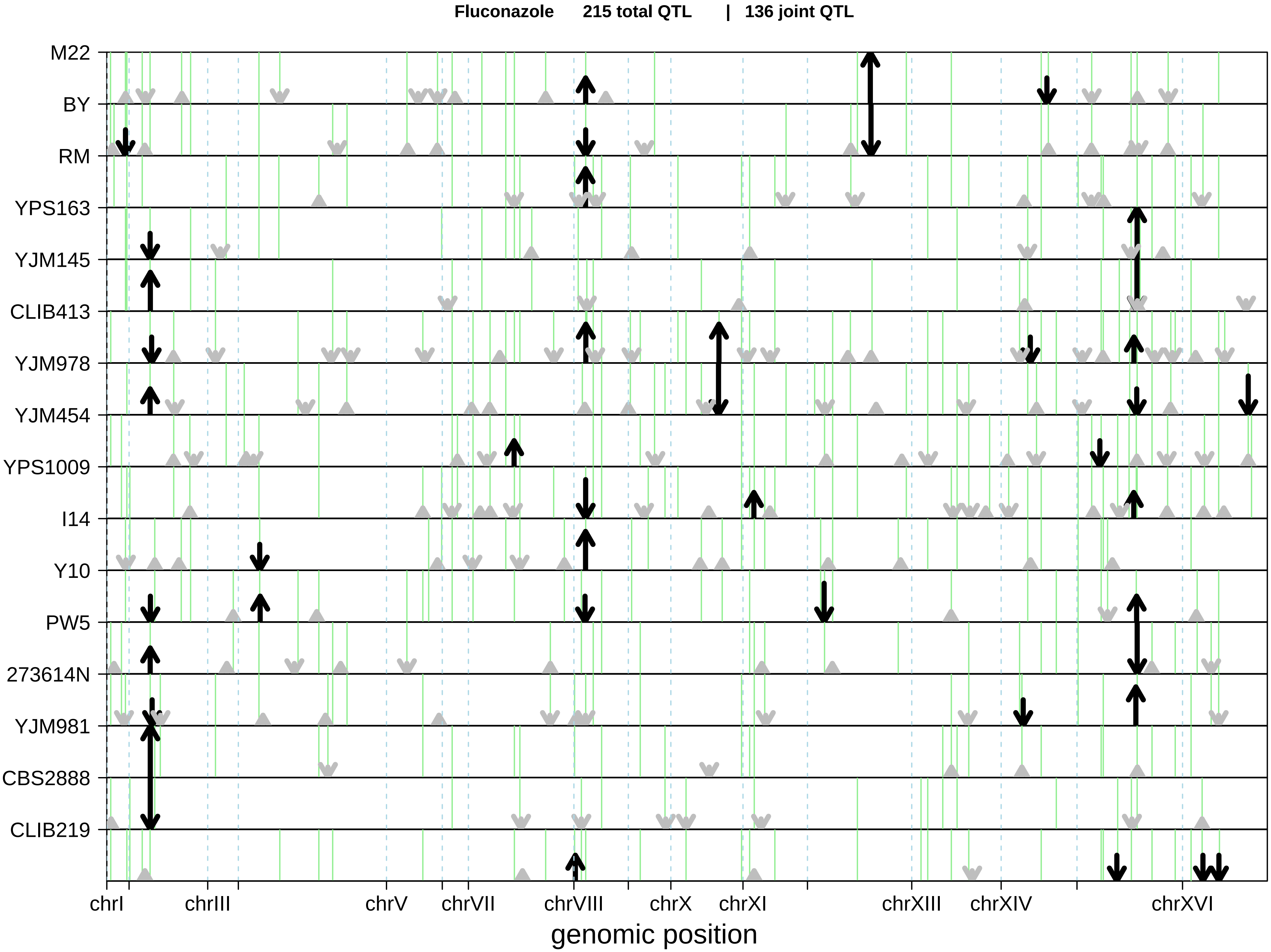

Fluconazole 215 total QTL | 136 joint QTL
M22
BY
RM
YPS163
YJM145
CLIB413
YJM978
YJM454
YPS1009
I14
Y10
PW5
273614N
YJM981
CBS2888
CLIB219
chrI
chrIII
chrV
chrVII
chrVIII
chrX
chrXI
chrXIII
chrXIV
chrXVI
genomic position

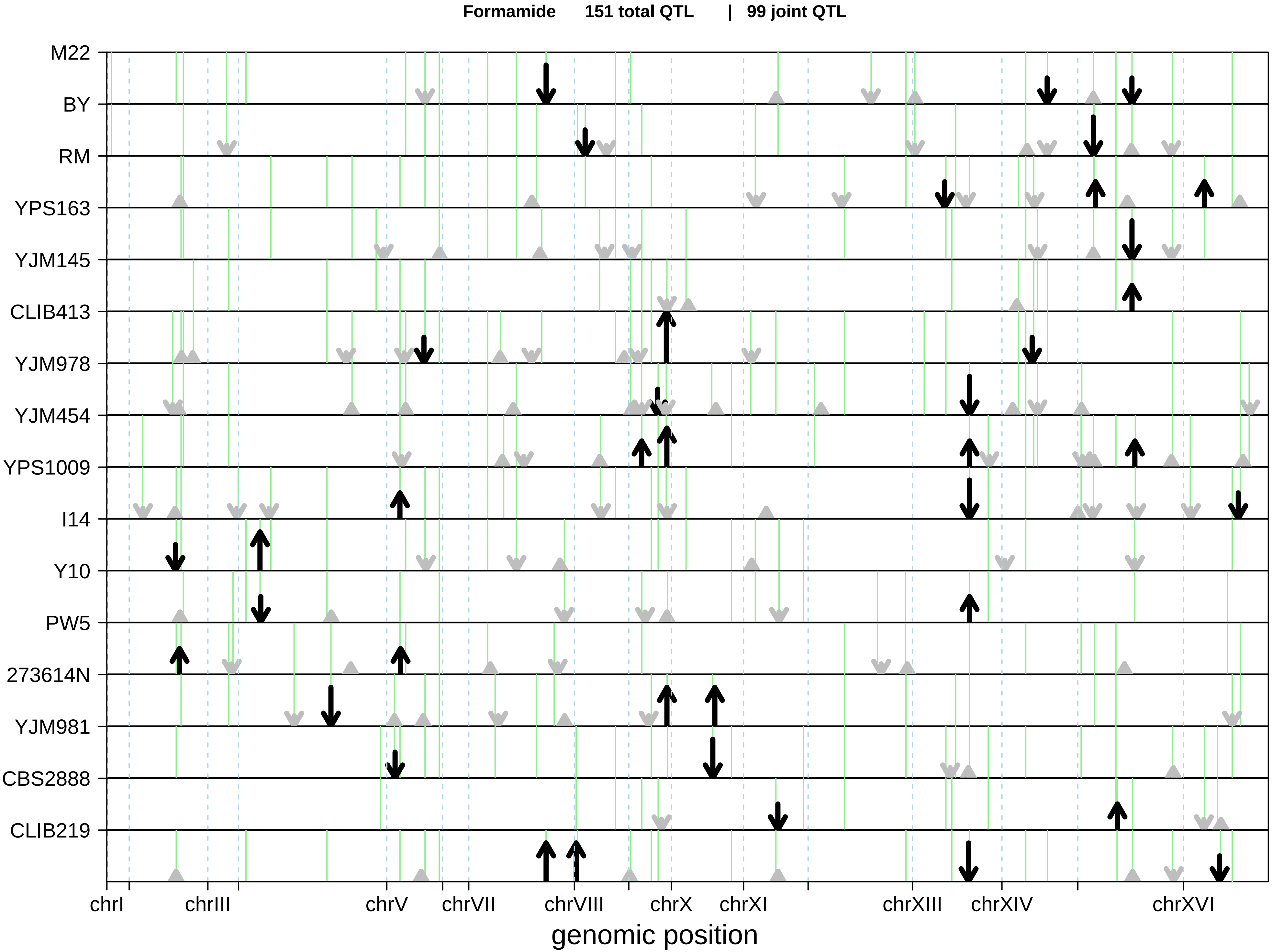
Formamide 151 total QTL | 99 joint QTL
M22
BY
RM
YPS163
YJM145
CLIB413
YJM978
YJM454
YPS1009
I14
Y10
PW5
273614N
YJM981
CBS2888
CLIB219
chrI
chrIII
chrV
chrVII
chrVIII
chrX
chrXI
chrXIII
chrXIV
chrXVI
genomic position

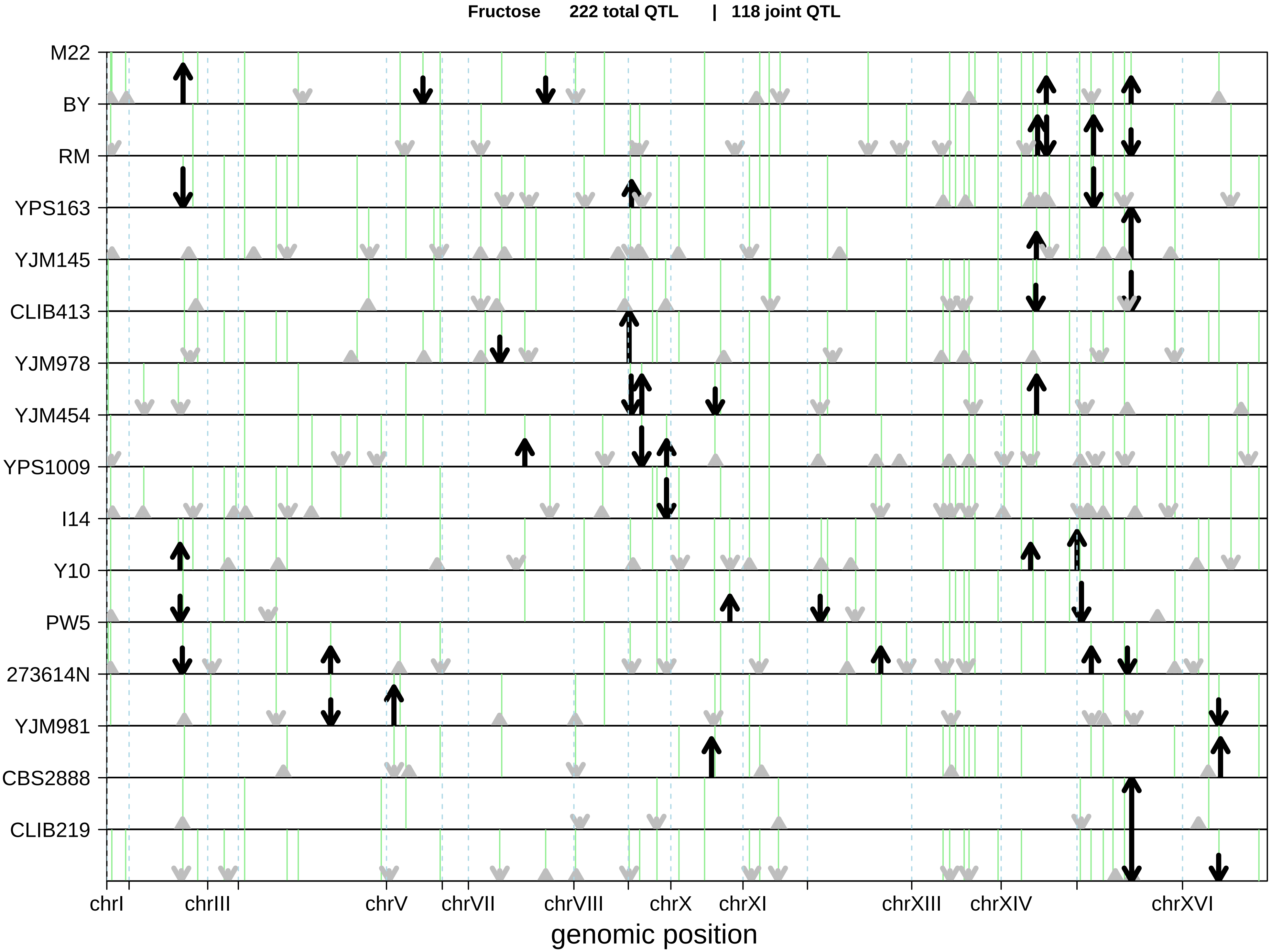
Fructose 222 total QTL | 118 joint QTL
M22
BY
RM
YPS163
YJM145
CLIB413
YJM978
YJM454
YPS1009
I14
Y10
PW5
273614N
YJM981
CBS2888
CLIB219
chrI
chrIII
chrV
chrVII
chrVIII
chrX
chrXI
chrXIII
chrXIV
chrXVI
genomic position

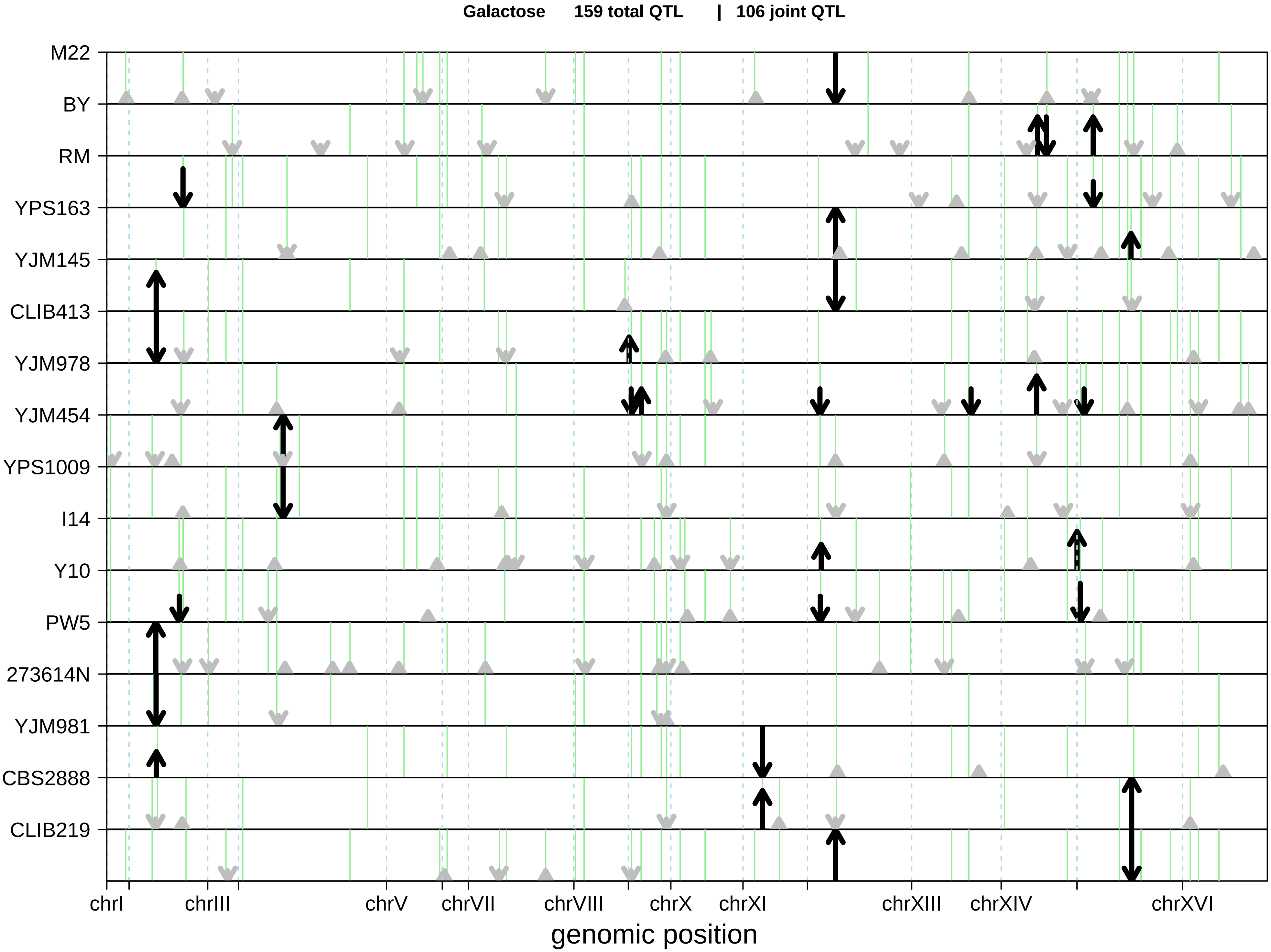
Galactose 159 total QTL | 106 joint QTL
M22
BY
RM
YPS163
YJM145
CLIB413
YJM978
YJM454
YPS1009
I14
Y10
PW5
273614N
YJM981
CBS2888
CLIB219
chrI
chrIII
chrV
chrVII
chrVIII
chrX
chrXI
chrXIII
chrXIV
chrXVI
genomic position

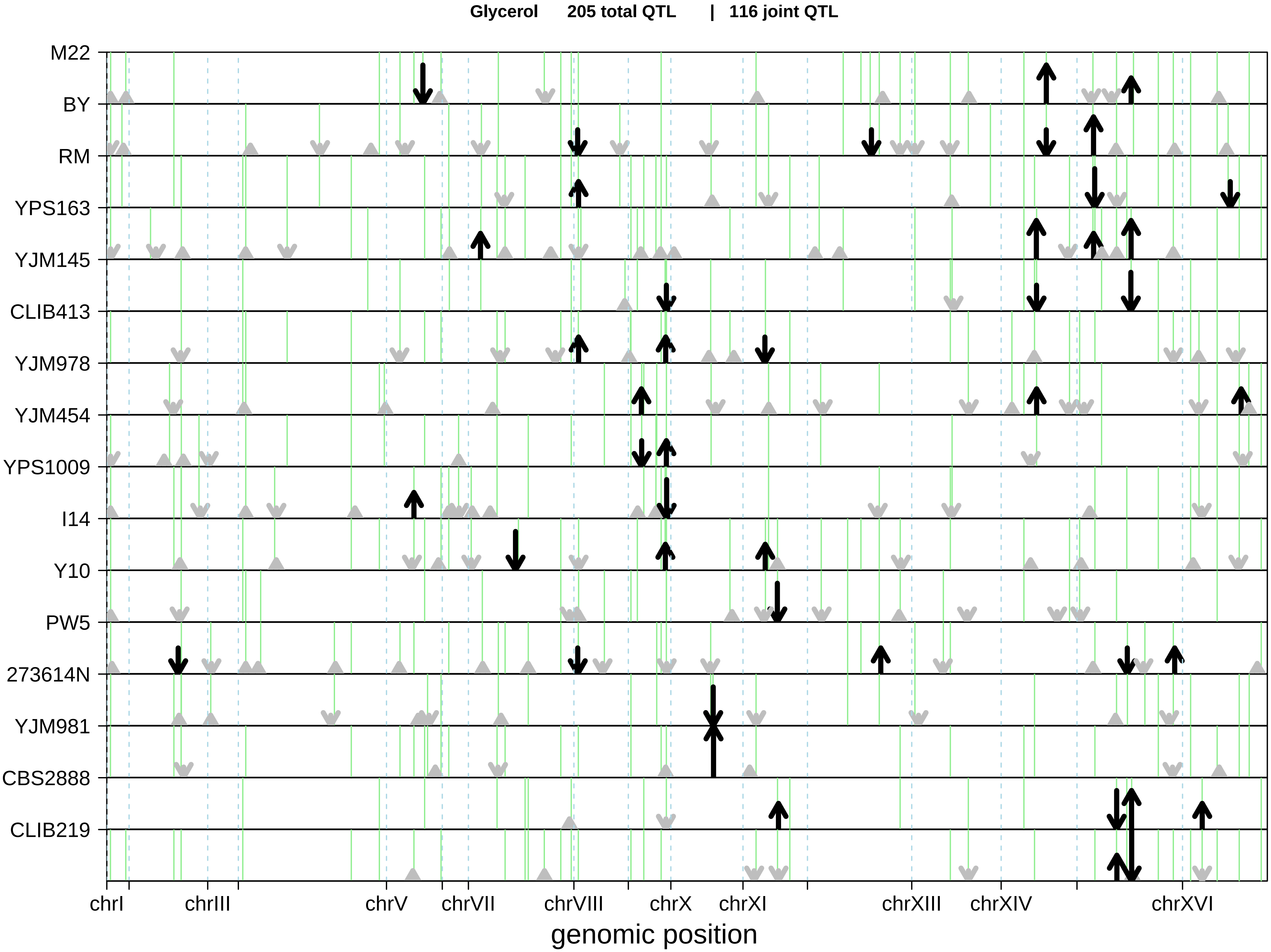

Glycerol 205 total QTL | 116 joint QTL
M22
BY
RM
YPS163
YJM145
CLIB413
YJM978
YJM454
YPS1009
I14
Y10
PW5
273614N
YJM981
CBS2888
CLIB219
chrI
chrIII
chrV
chrVII
chrVIII
chrX
chrXI
chrXIII
chrXIV
chrXVI
genomic position

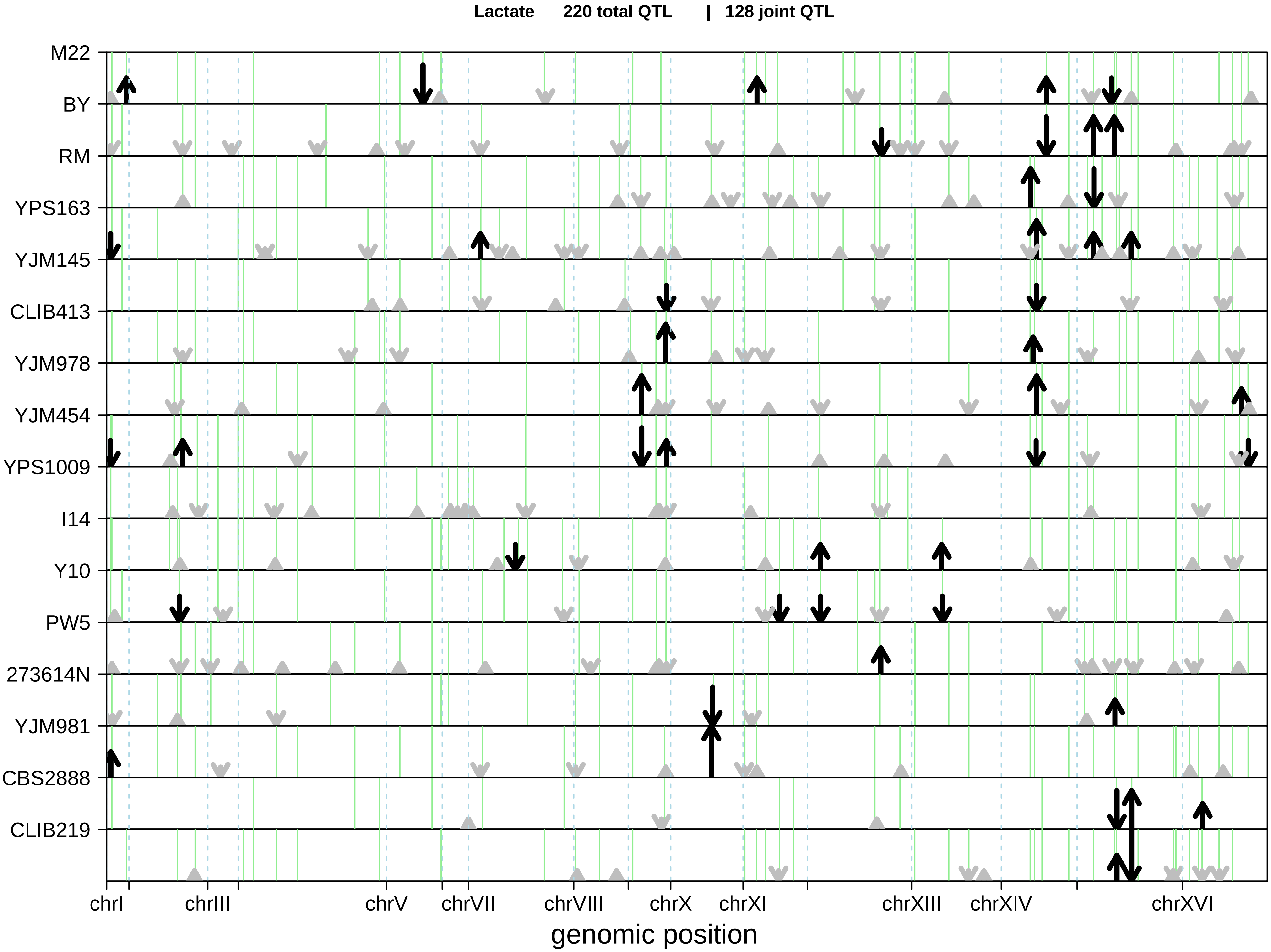
Lactate 220 total QTL | 128 joint QTL
M22
BY
RM
YPS163
YJM145
CLIB413
YJM978
YJM454
YPS1009
I14
Y10
PW5
273614N
YJM981
CBS2888
CLIB219
chrI
chrIII
chrV
chrVII
chrVIII
chrX
chrXI
chrXIII
chrXIV
chrXVI
genomic position

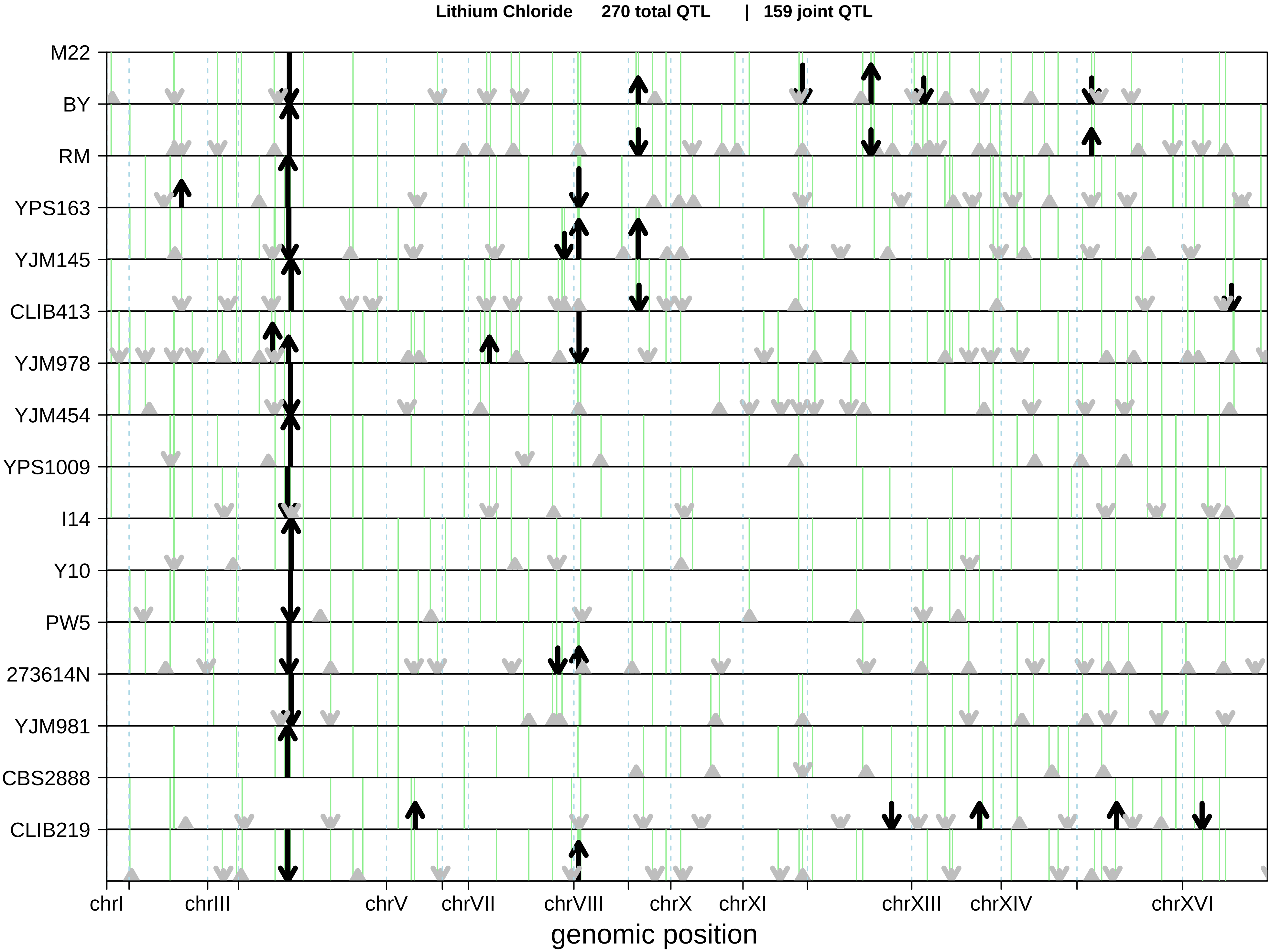
Lithium Chloride 270 total QTL | 159 joint QTL
M22
BY
RM
YPS163
YJM145
CLIB413
YJM978
YJM454
YPS1009
I14
Y10
PW5
273614N
YJM981
CBS2888
CLIB219
chrI
chrIII
chrV
chrVII
chrVIII
chrX
chrXI
chrXIII
chrXIV
chrXVI
genomic position

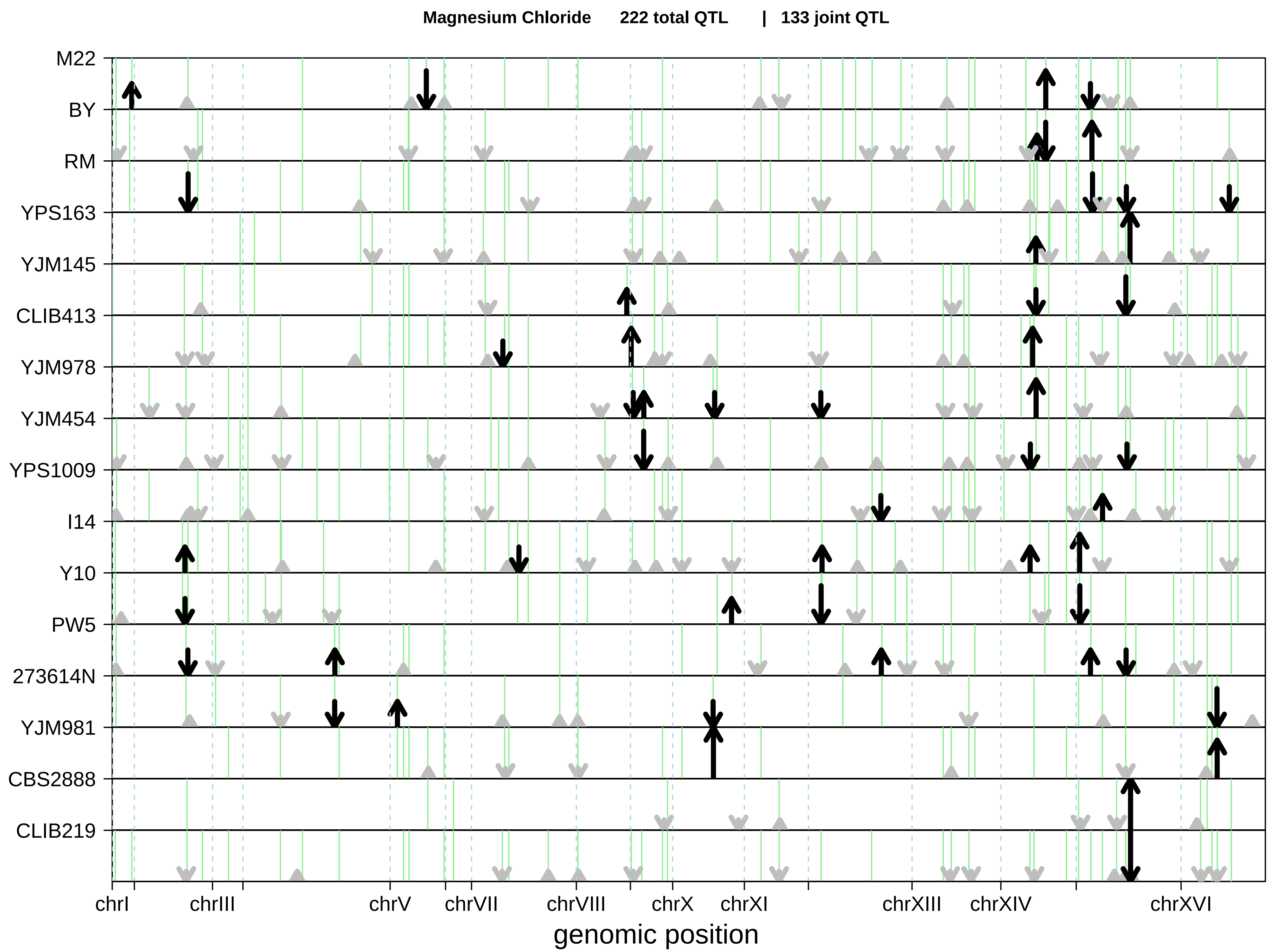
Magnesium Chloride 222 total QTL | 133 joint QTL
M22
BY
RM
YPS163
YJM145
CLIB413
YJM978
YJM454
YPS1009
I14
Y10
PW5
273614N
YJM981
CBS2888
CLIB219
chrI
chrIII
chrV
chrVII
chrVIII
chrX
chrXI
chrXIII
chrXIV
chrXVI
genomic position

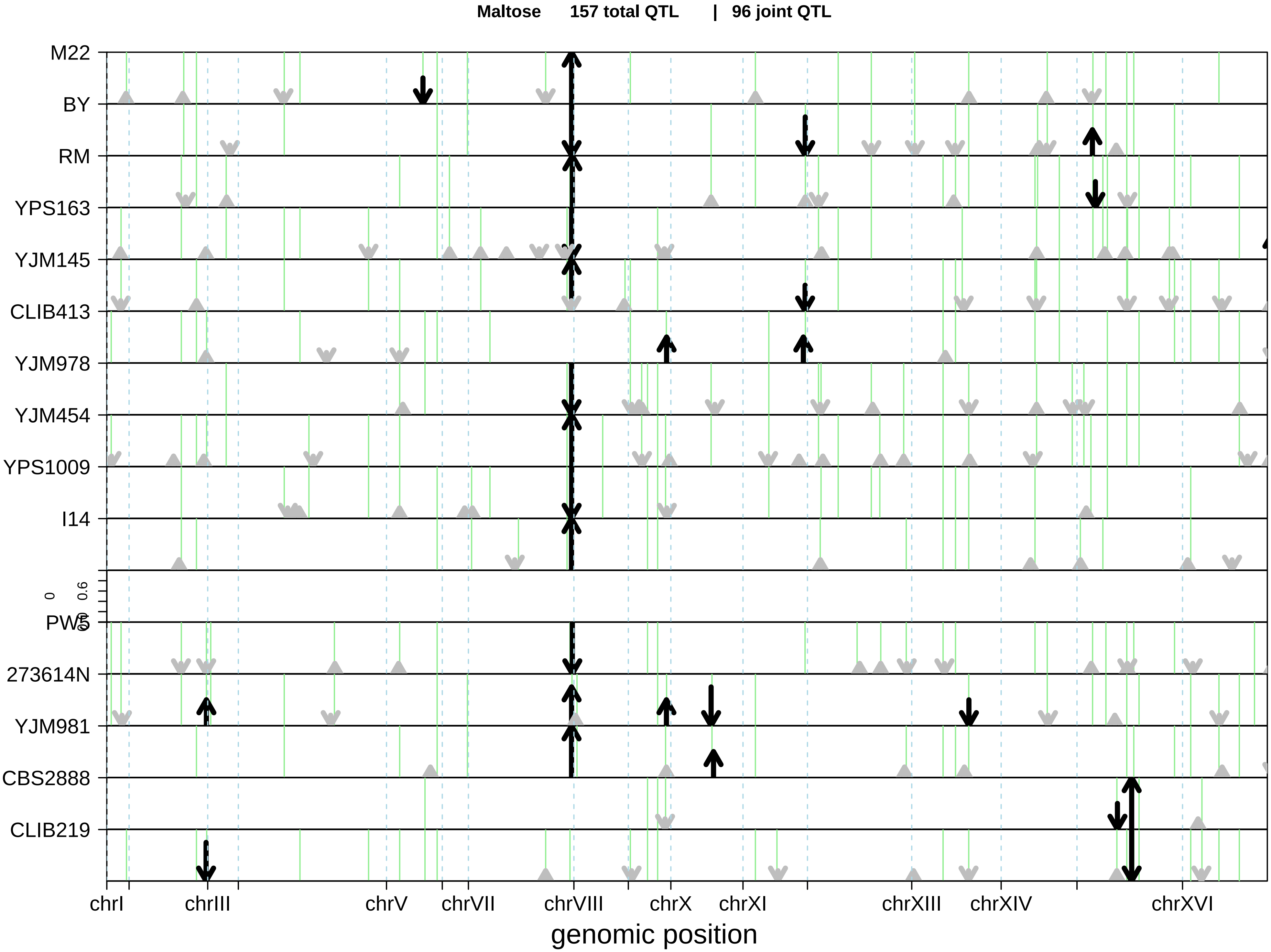
Maltose 157 total QTL | 96 joint QTL
M22
BY
RM
YPS163
YJM145
CLIB413
YJM978
YJM454
YPS1009
I14
0
0.6
PW5
273614N
YJM981
CBS2888
CLIB219
chrI
chrIII
chrV
chrVII
chrVIII
chrX
chrXI
chrXIII
chrXIV
chrXVI
genomic position

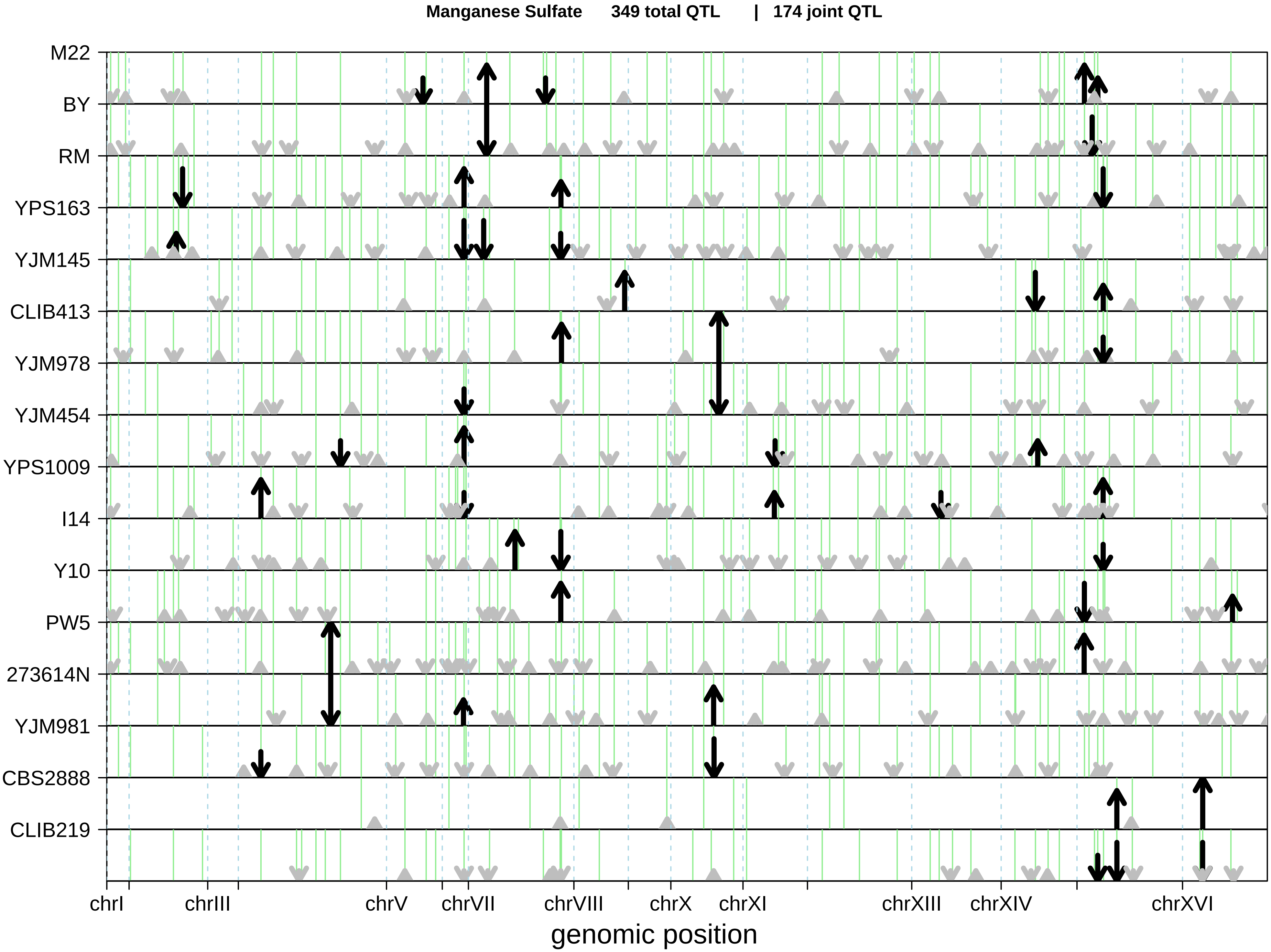

Manganese Sulfate
349 total QTL
| 174 joint QTL
M22
BY
RM
YPS163
YJM145
CLIB413
YJM978
YJM454
YPS1009
I14
Y10
PW5
273614N
YJM981
CBS2888
CLIB219
chrI
chrIII
chrV
chrVII
chrVIII
chrX
chrXI
chrXIII
chrXIV
chrXVI
genomic position

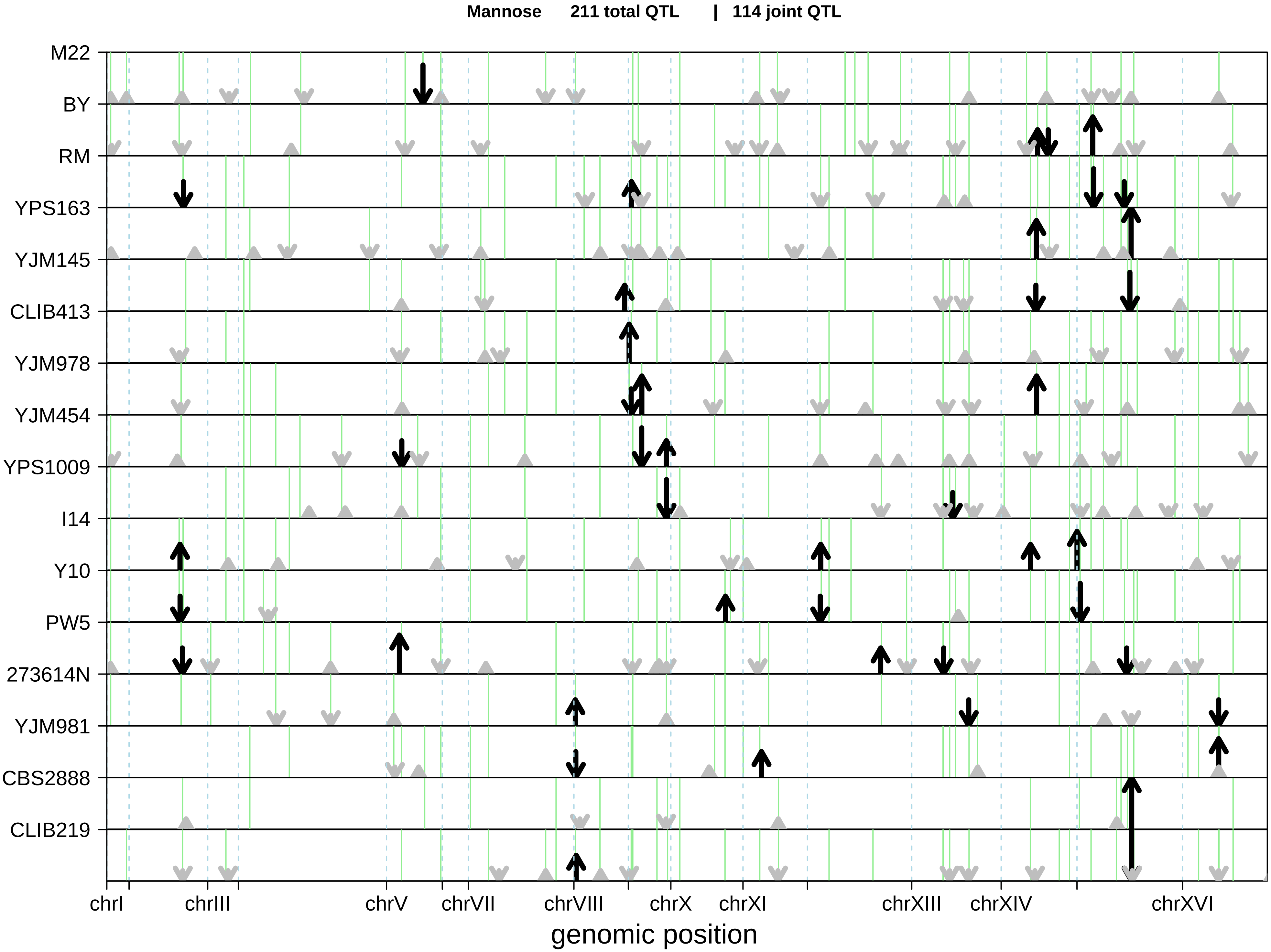

Mannose 211 total QTL | 114 joint QTL
M22
BY
RM
YPS163
YJM145
CLIB413
YJM978
YJM454
YPS1009
I14
Y10
PW5
273614N
YJM981
CBS2888
CLIB219
chrI
chrIII
chrV
chrVII
chrVIII
chrX
chrXI
chrXIII
chrXIV
chrXVI
genomic position

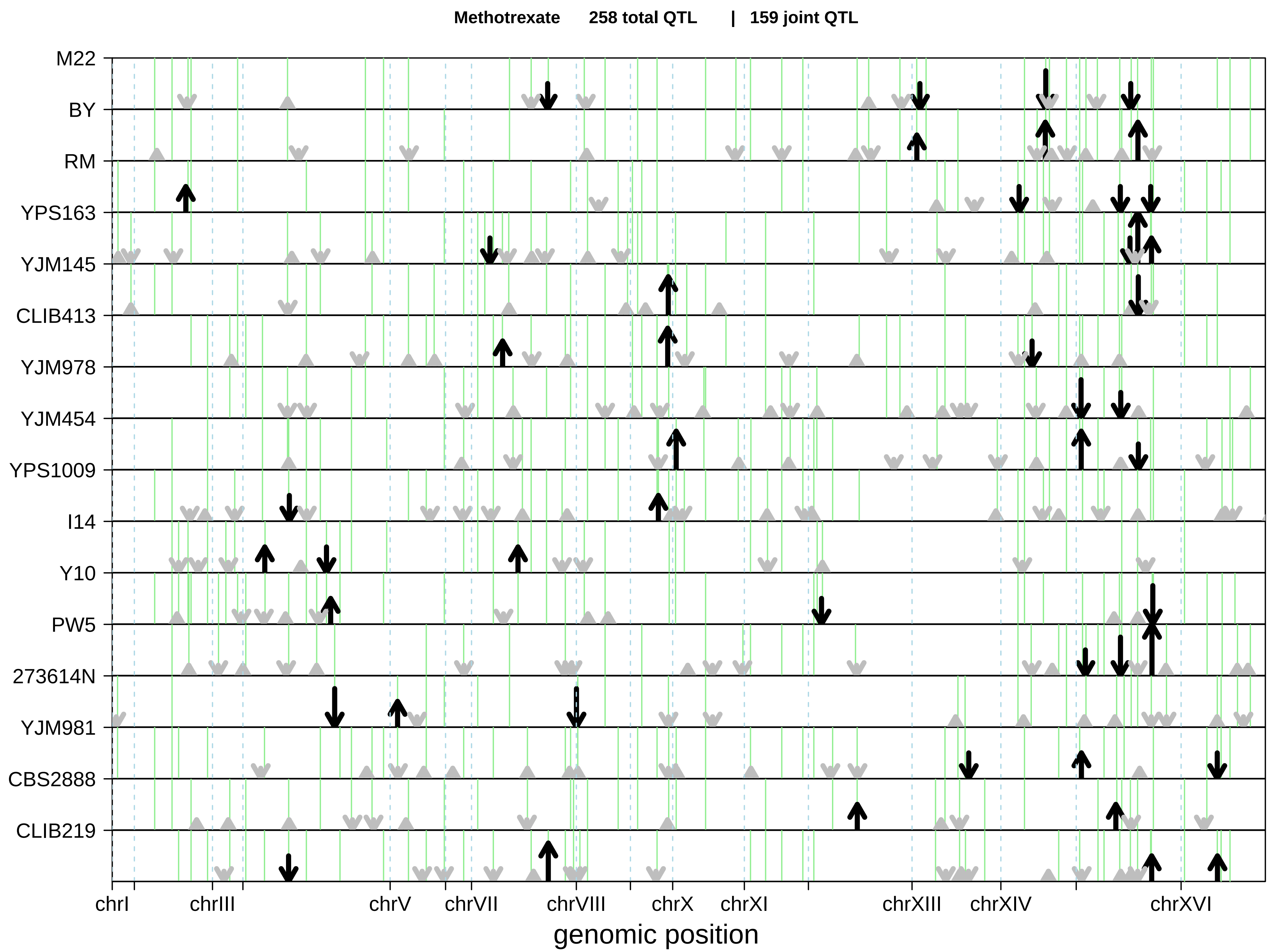
Methotrexate 258 total QTL | 159 joint QTL
M22
BY
RM
YPS163
YJM145
CLIB413
YJM978
YJM454
YPS1009
I14
Y10
PW5
273614N
YJM981
CBS2888
CLIB219
chrI
chrIII
chrV
chrVII
chrVIII
chrX
chrXI
chrXIII
chrXIV
chrXVI
genomic position

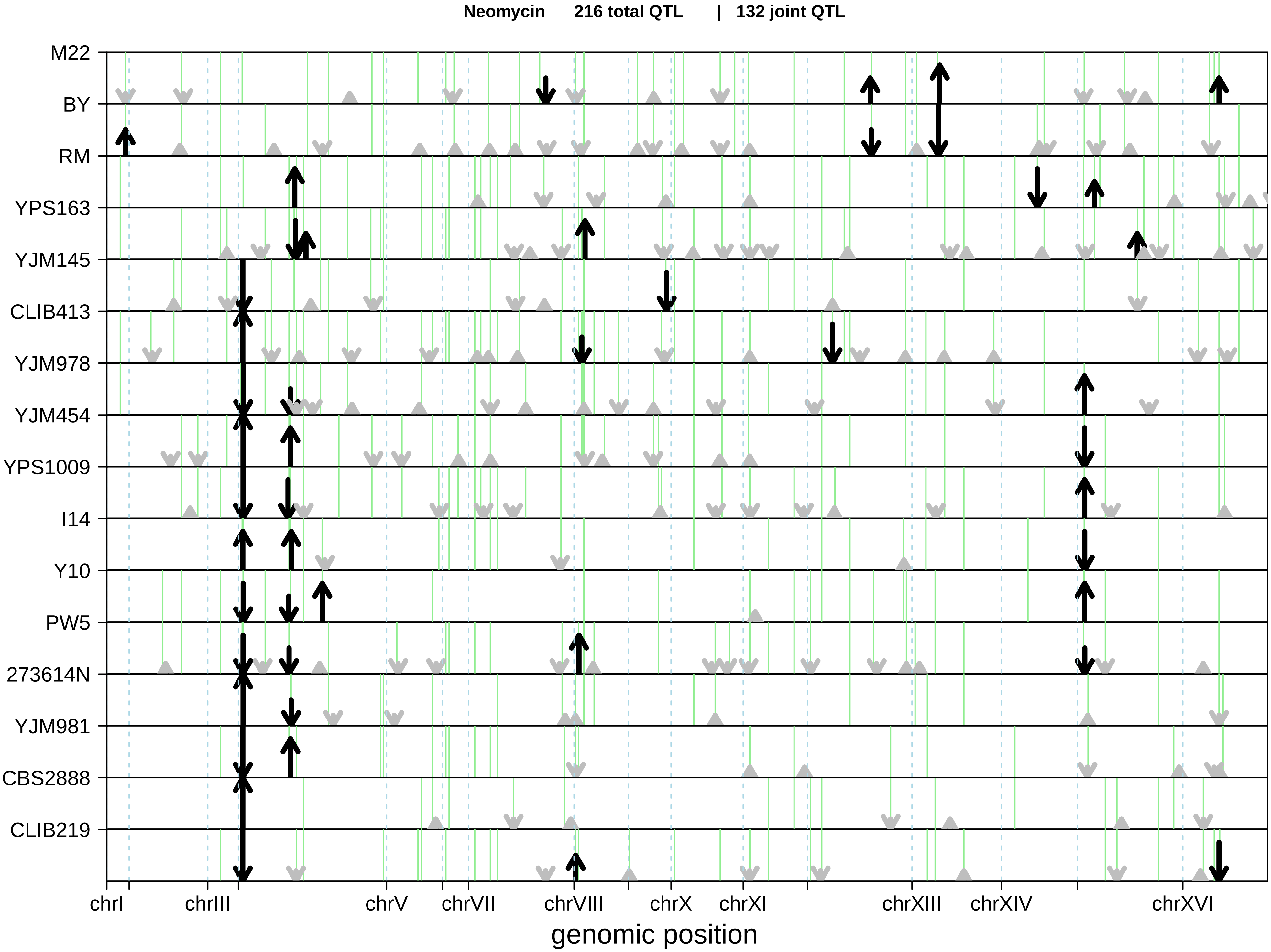

Neomycin 216 total QTL | 132 joint QTL
M22
BY
RM
YPS163
YJM145
CLIB413
YJM978
YJM454
YPS1009
I14
Y10
PW5
273614N
YJM981
CBS2888
CLIB219
chrI
chrIII
chrV
chrVII
chrVIII
chrX
chrXI
chrXIII
chrXIV
chrXVI
genomic position

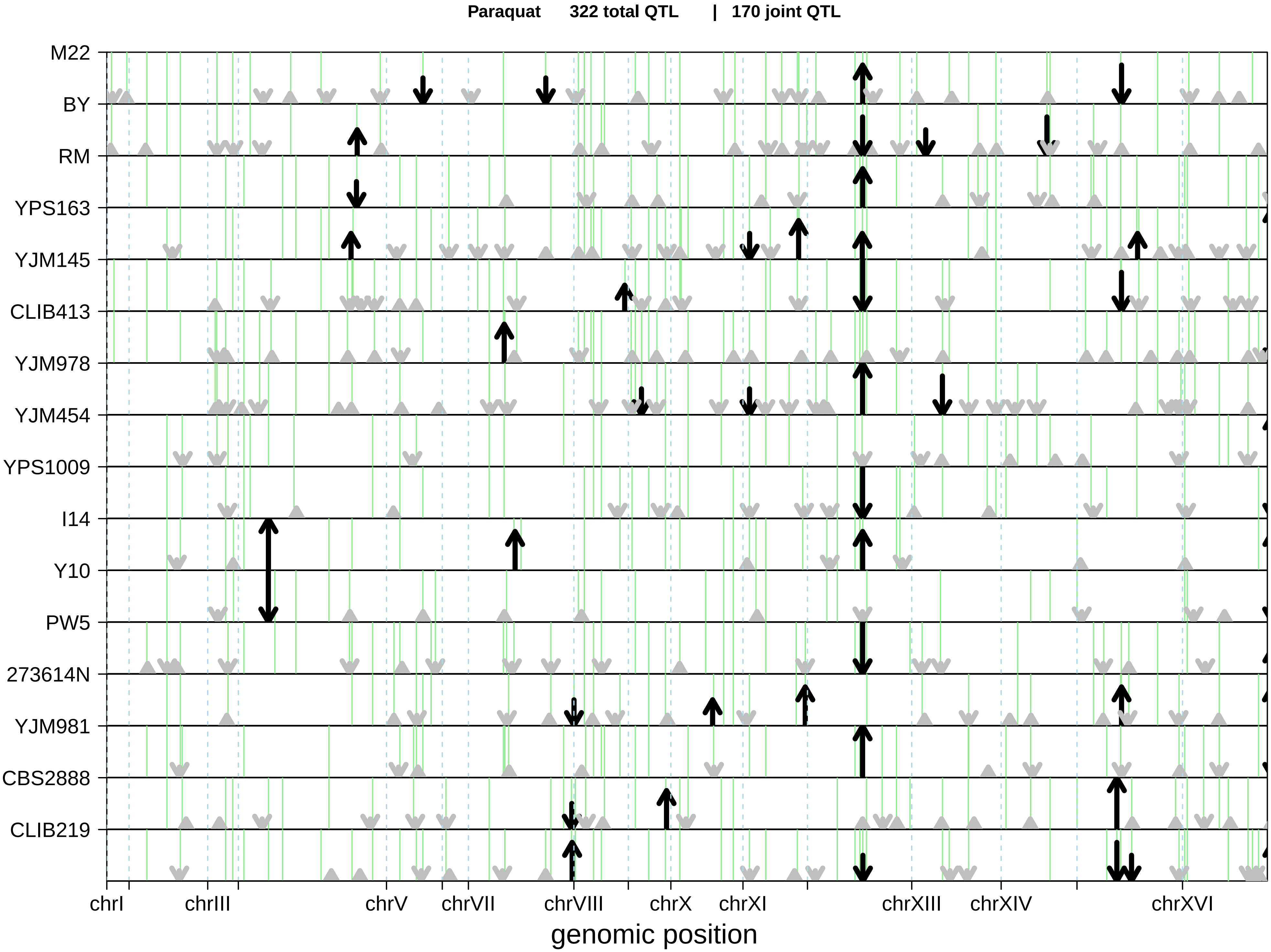

Paraquat 322 total QTL | 170 joint QTL
M22
BY
RM
YPS163
YJM145
CLIB413
YJM978
YJM454
YPS1009
I14
Y10
PW5
273614N
YJM981
CBS2888
CLIB219
chrI
chrIII
chrV
chrVII
chrVIII
chrX
chrXI
chrXIII
chrXIV
chrXVI
genomic position

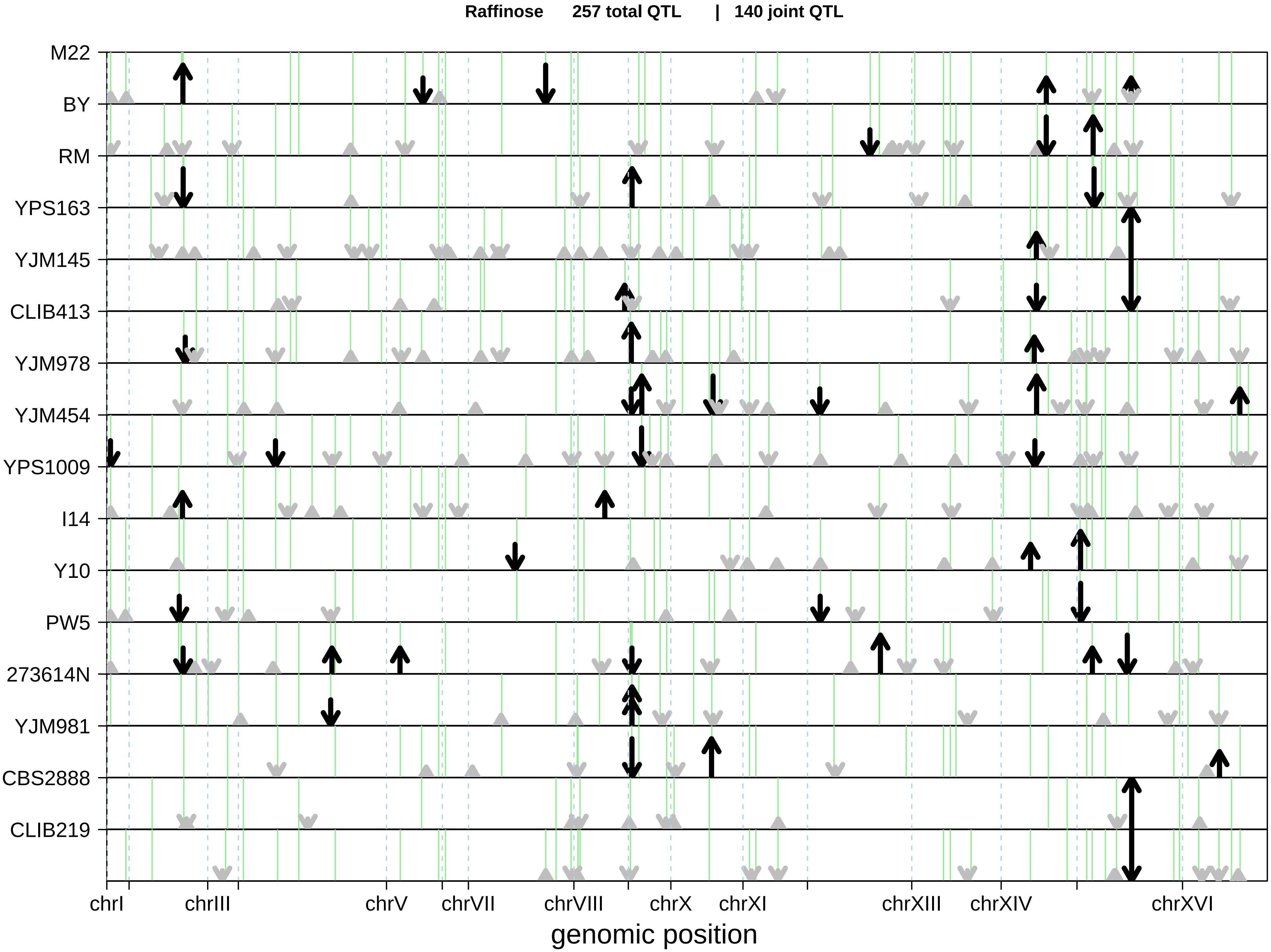

Raffinose 257 total QTL | 140 joint QTL
M22
BY
RM
YPS163
YJM145
CLIB413
YJM978
YJM454
YPS1009
I14
Y10
PW5
273614N
YJM981
CBS2888
CLIB219
chrI
chrIII
chrV
chrVII
chrVIII
chrX
chrXI
chrXIII
chrXIV
chrXVI
genomic position

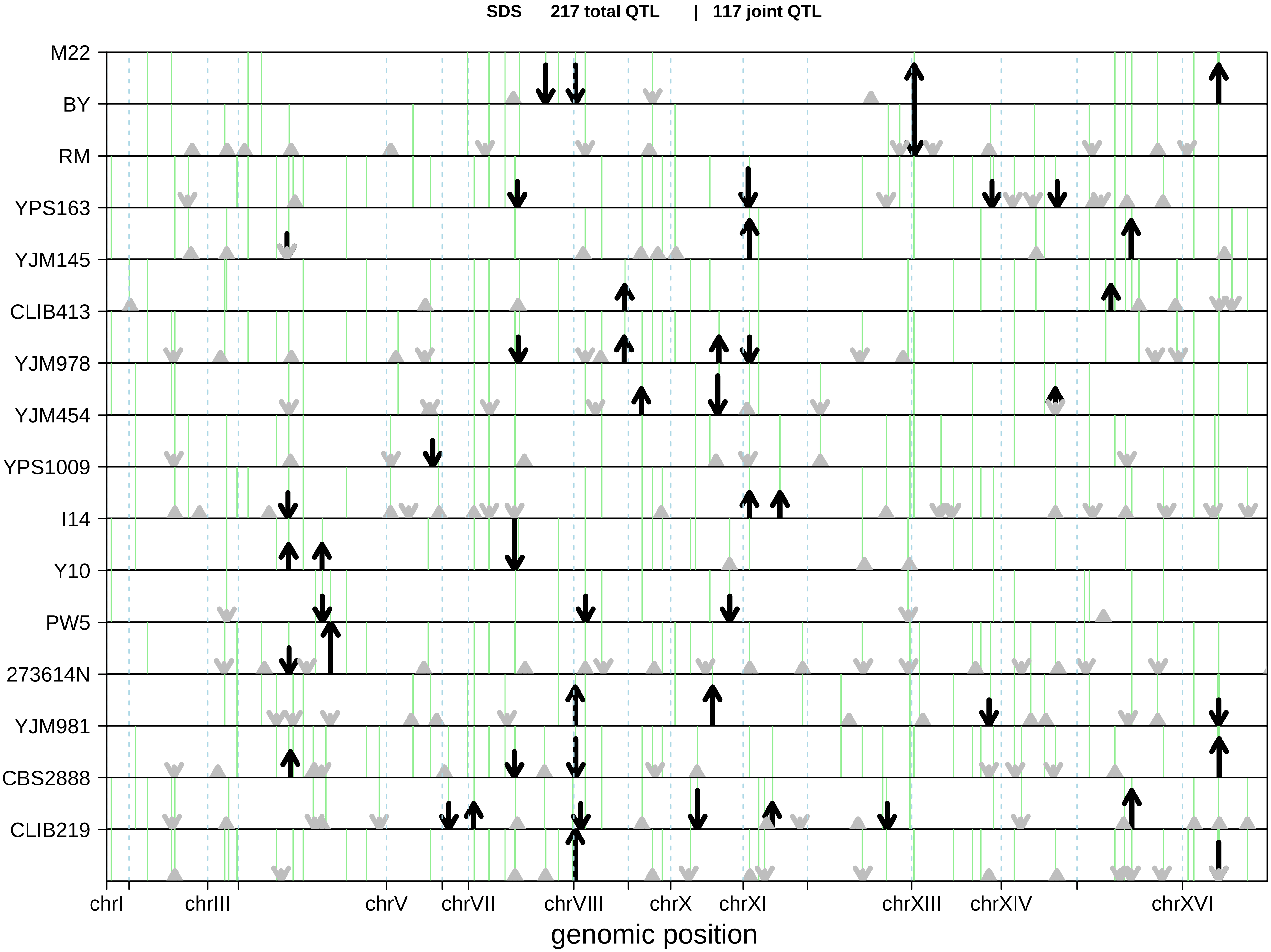

SDS 217 total QTL | 117 joint QTL
M22
BY
RM
YPS163
YJM145
CLIB413
YJM978
YJM454
YPS1009
I14
Y10
PW5
273614N
YJM981
CBS2888
CLIB219
chrI
chrIII
chrV
chrVII
chrVIII
chrX
chrXI
chrXIII
chrXIV
chrXVI
genomic position

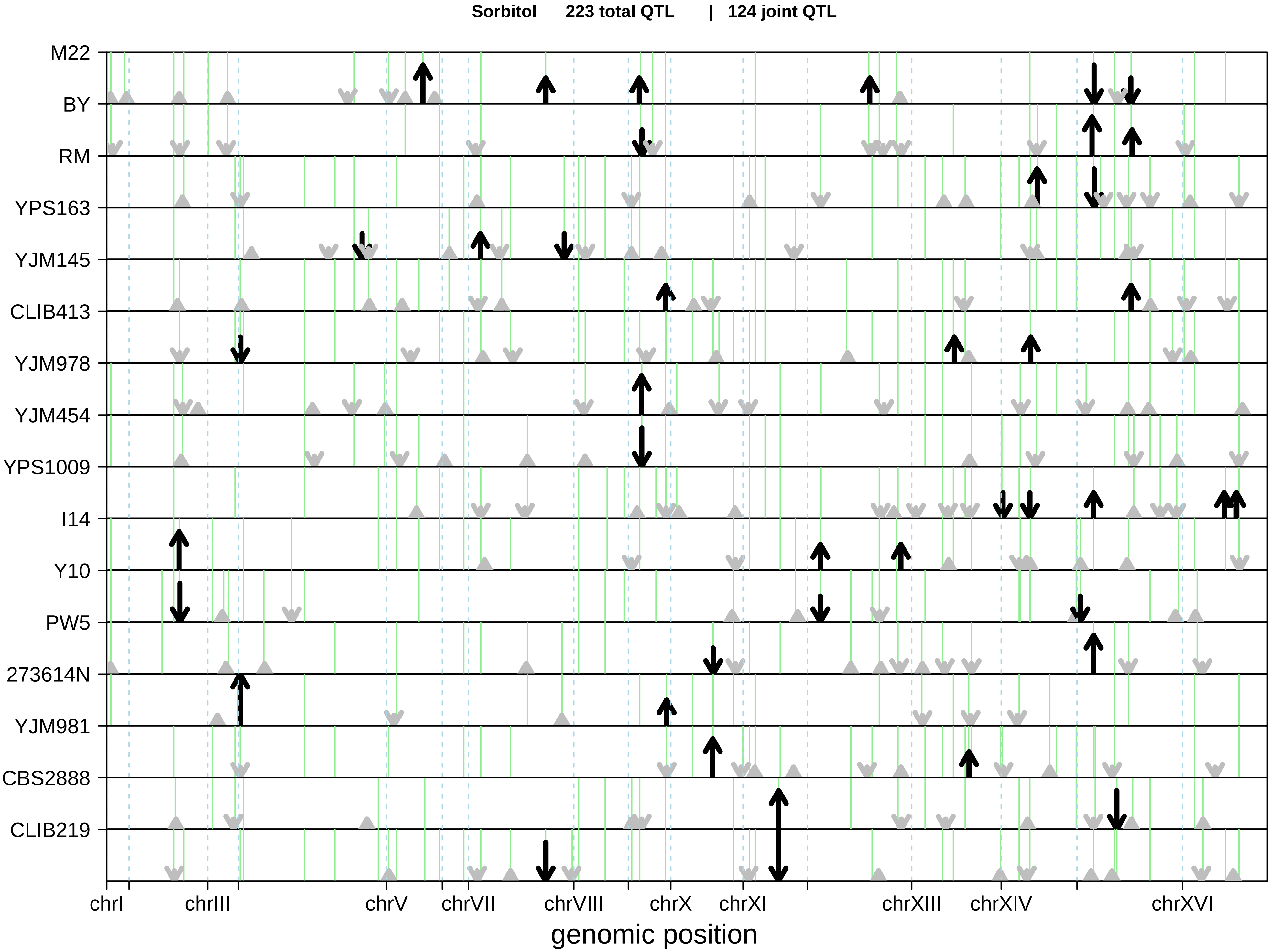
Sorbitol 223 total QTL | 124 joint QTL
M22
BY
RM
YPS163
YJM145
CLIB413
YJM978
YJM454
YPS1009
I14
Y10
PW5
273614N
YJM981
CBS2888
CLIB219
chrI
chrIII
chrV
chrVII
chrVIII
chrX
chrXI
chrXIII
chrXIV
chrXVI
genomic position

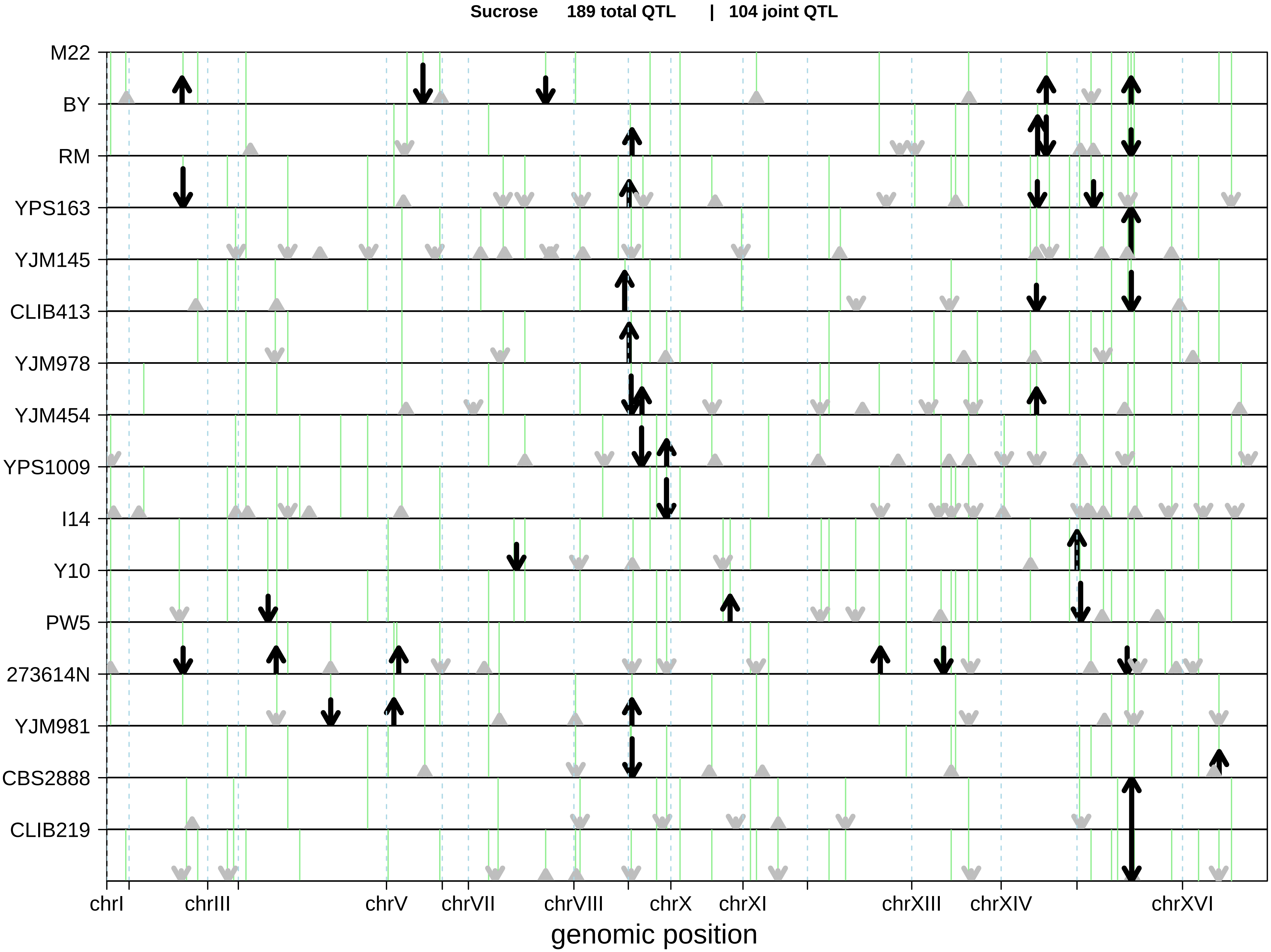
Sucrose 189 total QTL | 104 joint QTL
M22
BY
RM
YPS163
YJM145
CLIB413
YJM978
YJM454
YPS1009
I14
Y10
PW5
273614N
YJM981
CBS2888
CLIB219
chrI
chrIII
chrV
chrVII
chrVIII
chrX
chrXI
chrXIII
chrXIV
chrXVI
genomic position

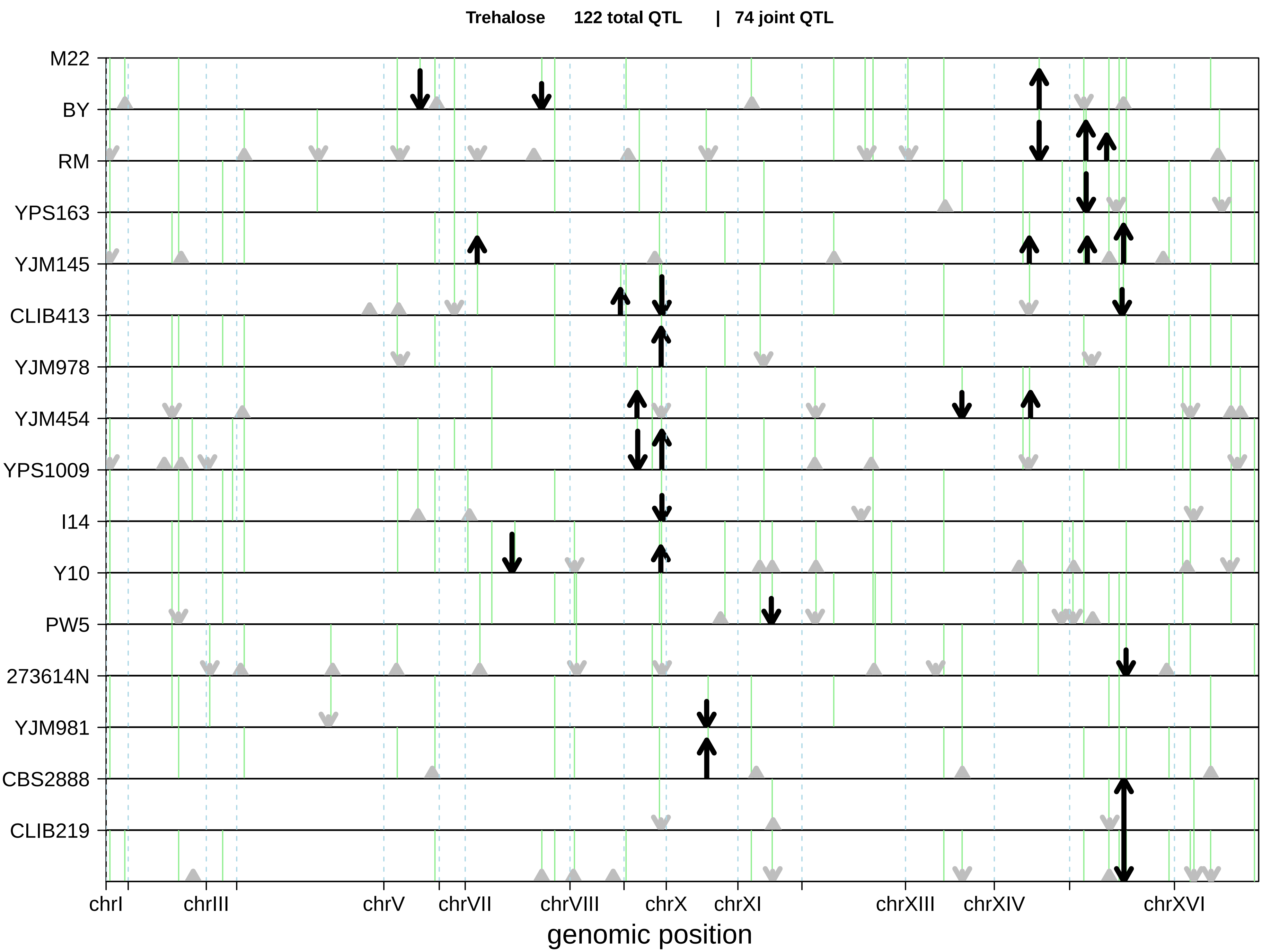
Trehalose 122 total QTL | 74 joint QTL
M22
BY
RM
YPS163
YJM145
CLIB413
YJM978
YJM454
YPS1009
I14
Y10
PW5
273614N
YJM981
CBS2888
CLIB219
chrI
chrIII
chrV
chrVII
chrVIII
chrX
chrXI
chrXIII
chrXIV
chrXVI
genomic position

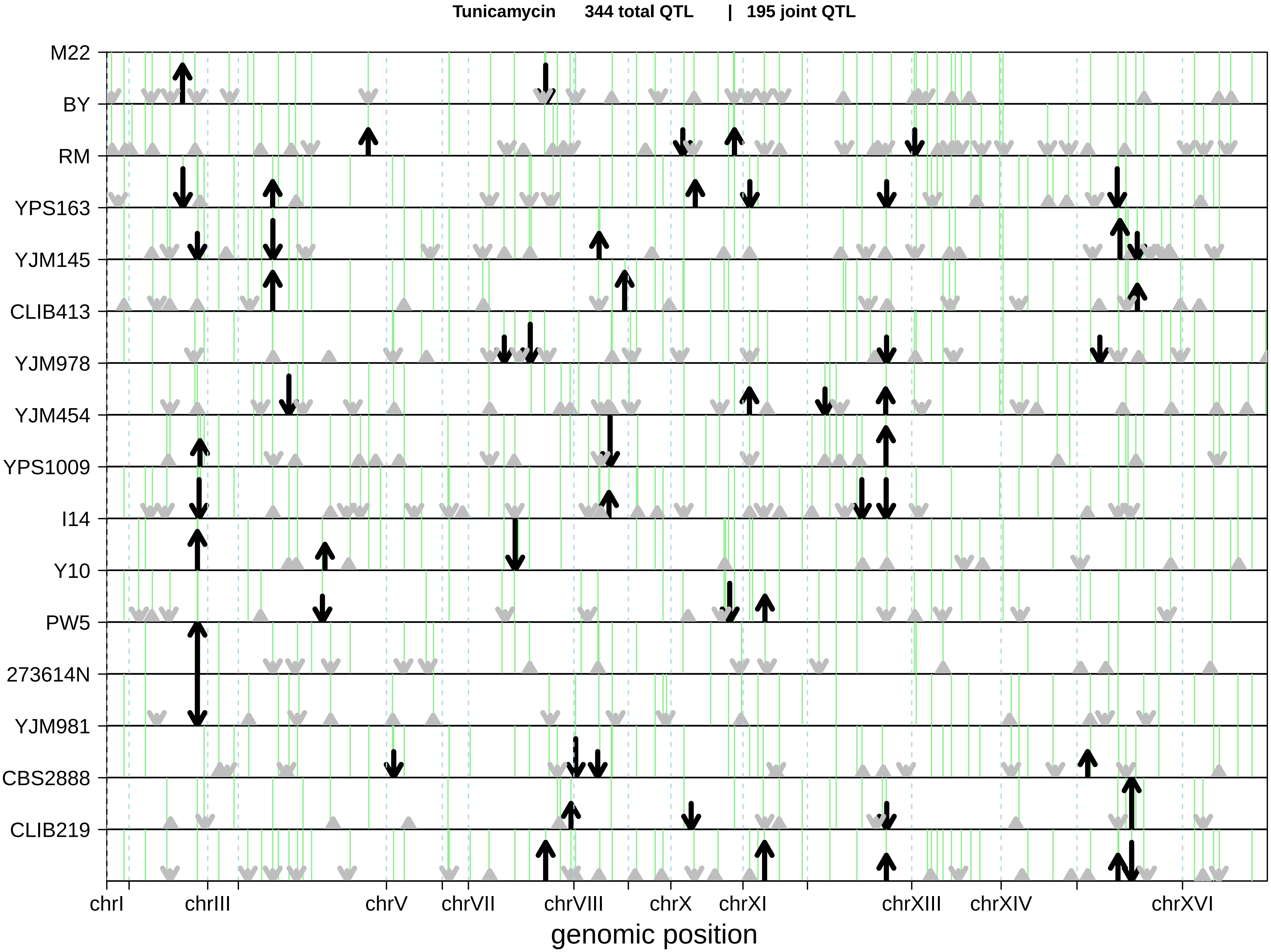

Tunicamycin 344 total QTL | 195 joint QTL
M22
BY
RM
YPS163
YJM145
CLIB413
YJM978
YJM454
YPS1009
I14
Y10
PW5
273614N
YJM981
CBS2888
CLIB219
chrI
chrIII
chrV
chrVII
chrVIII
chrX
chrXI
chrXIII
chrXIV
chrXVI
genomic position

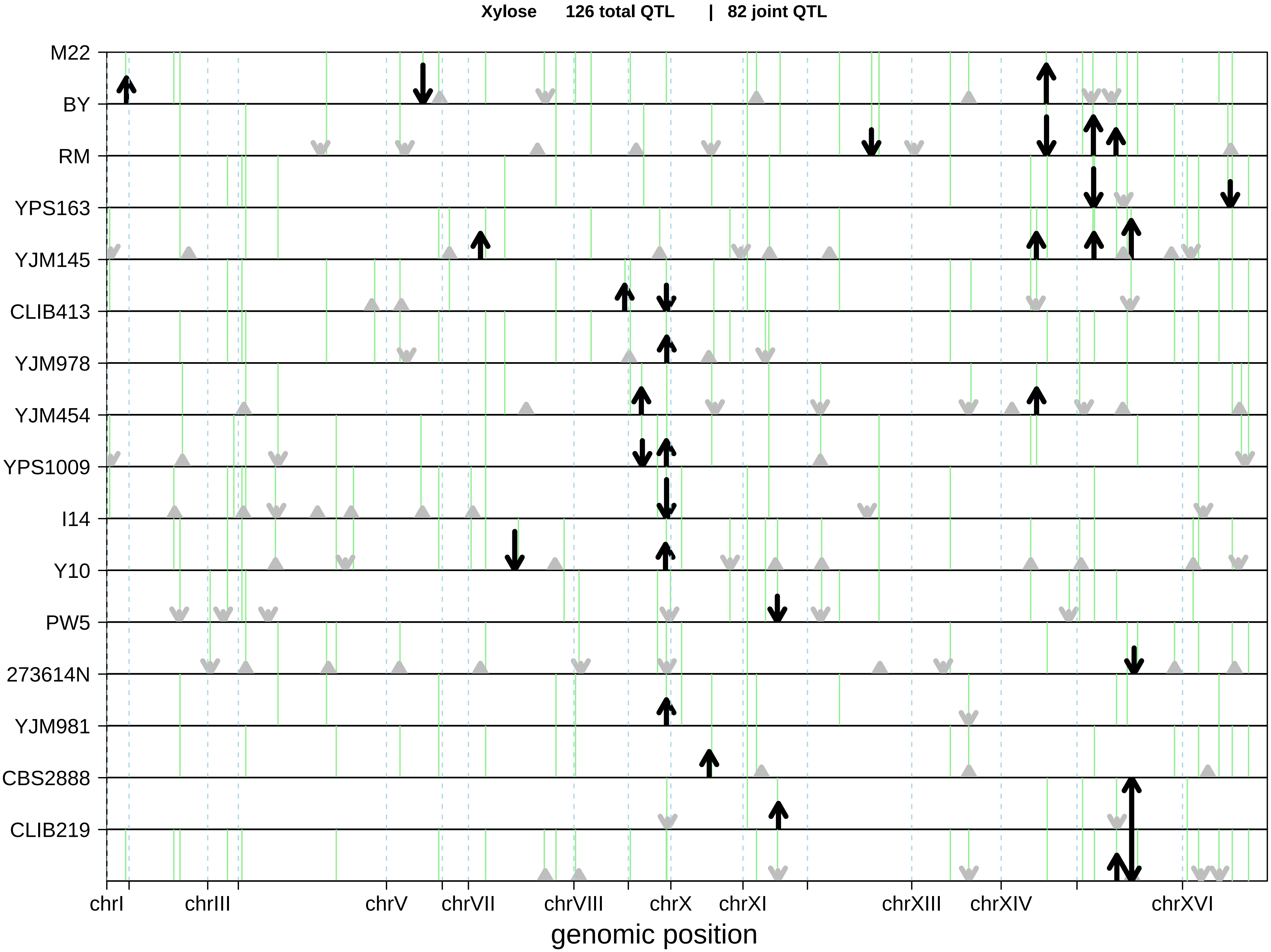
Xylose 126 total QTL | 82 joint QTL
M22
BY
RM
YPS163
YJM145
CLIB413
YJM978
YJM454
YPS1009
I14
Y10
PW5
273614N
YJM981
CBS2888
CLIB219
chrI
chrIII
chrV
chrVII
chrVIII
chrX
chrXI
chrXIII
chrXIV
chrXVI
genomic position

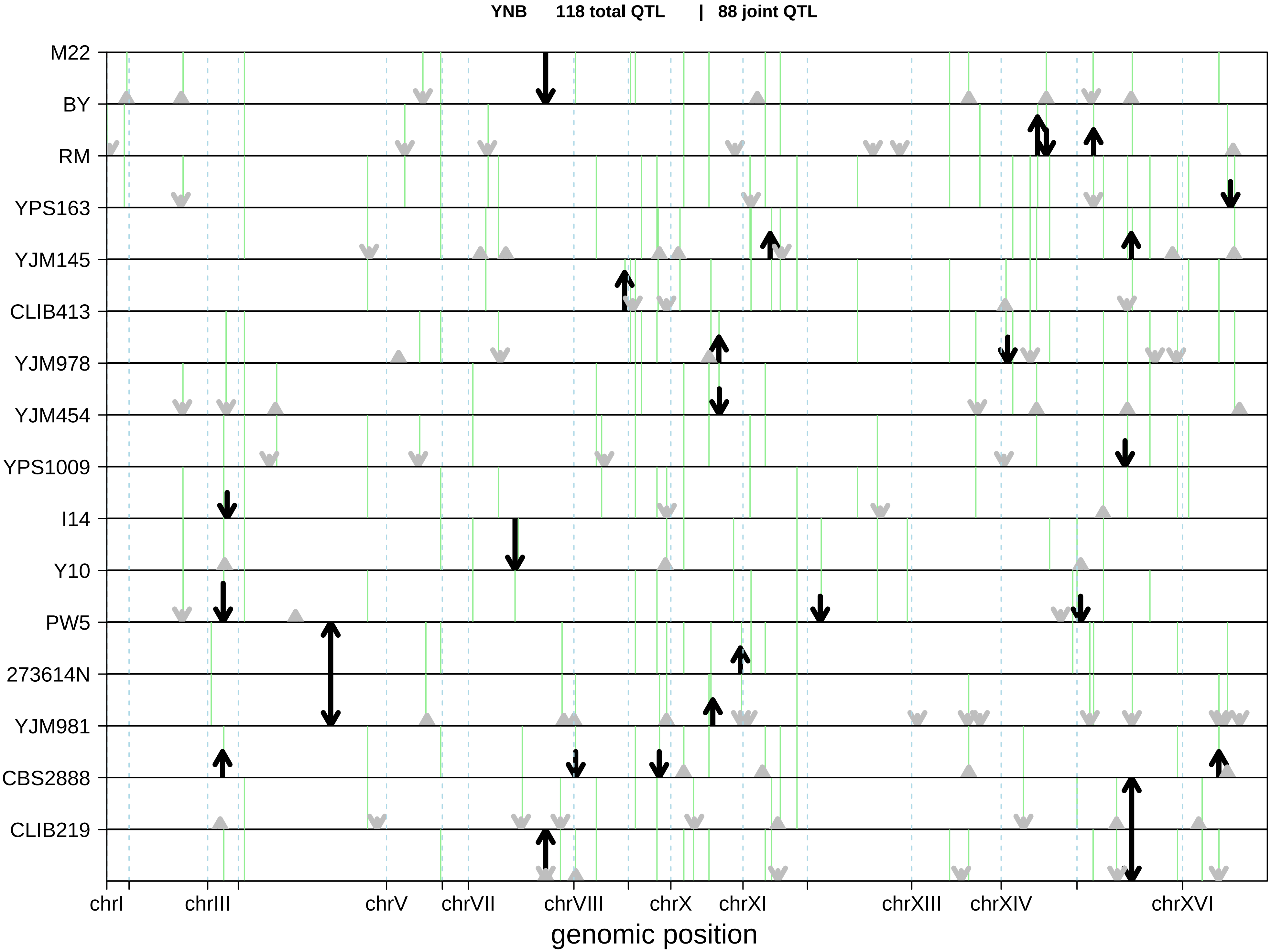
YNB 118 total QTL | 88 joint QTL
M22
BY
RM
YPS163
YJM145
CLIB413
YJM978
YJM454
YPS1009
I14
Y10
PW5
273614N
YJM981
CBS2888
CLIB219
chrI
chrIII
chrV
chrVII
chrVIII
chrX
chrXI
chrXIII
chrXIV
chrXVI
genomic position

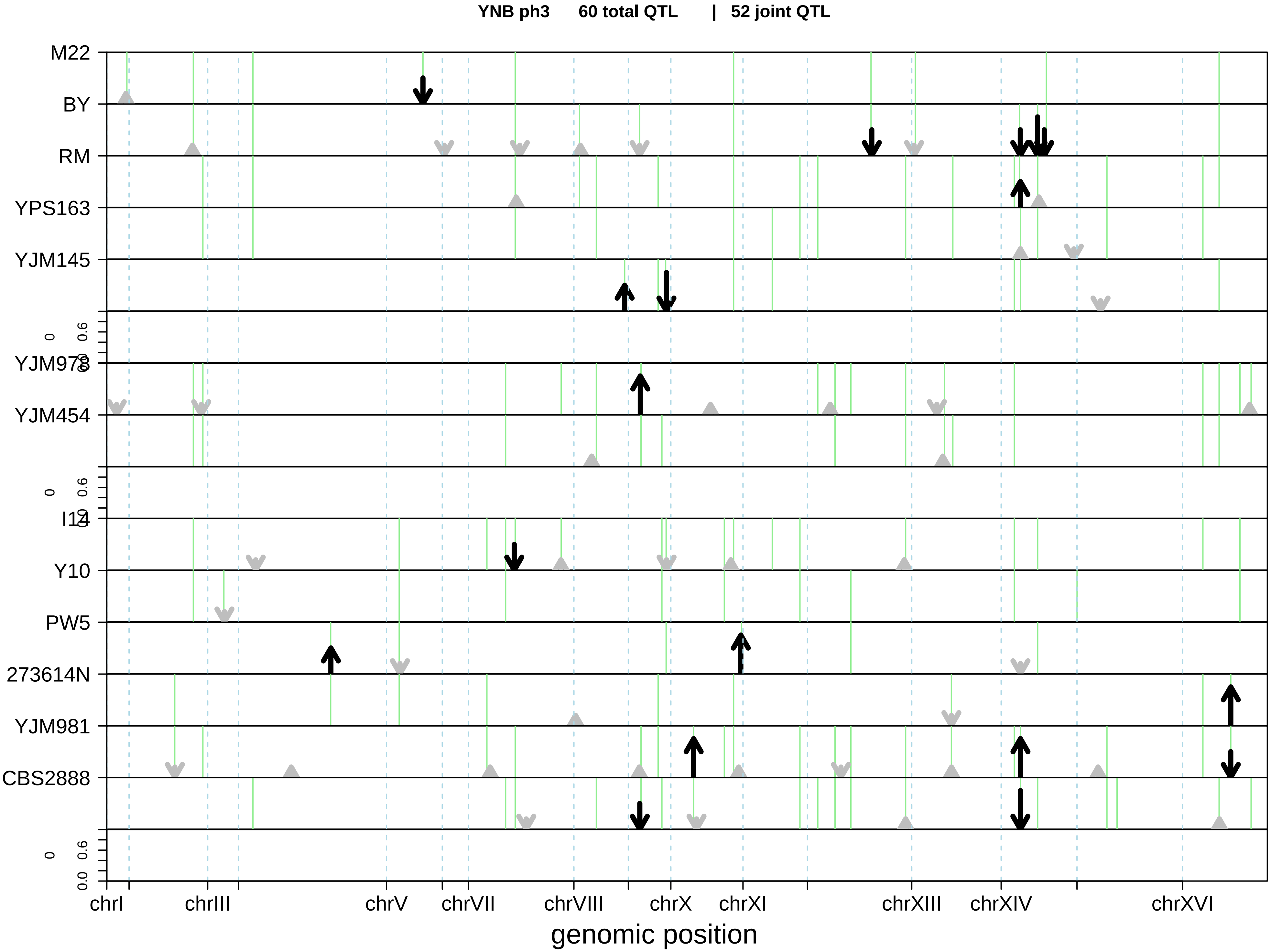
YNB ph3 60 total QTL | 52 joint QTL
M22
BY
RM
YPS163
YJM145
YJM978
YJM454
I14
Y10
PW5
273614N
YJM981
CBS2888
0
0.6
0.0
chrI
chrIII
chrV
chrVII
chrVIII
chrX
chrXI
chrXIII
chrXIV
chrXVI
genomic position

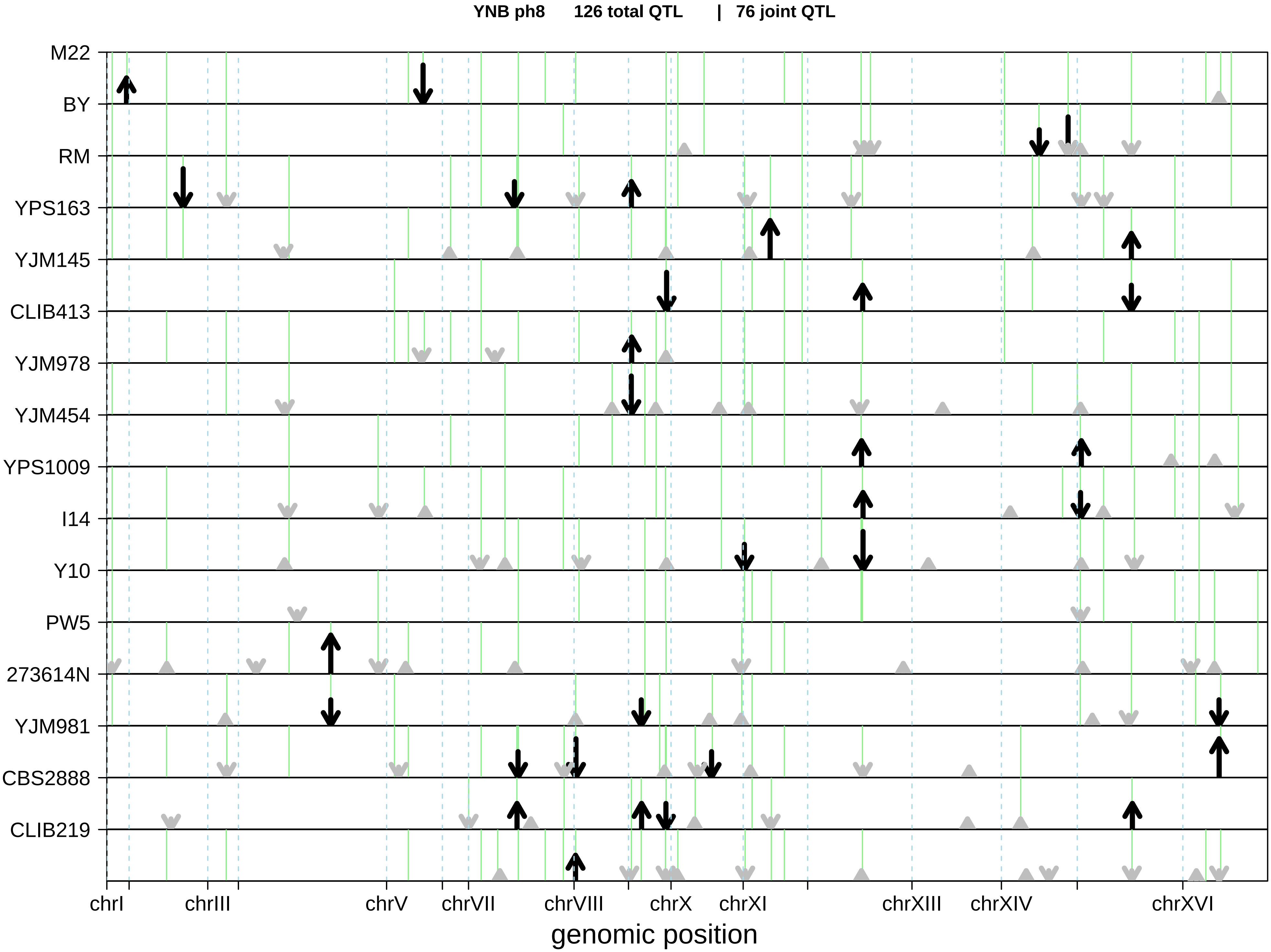

YNB ph8 126 total QTL | 76 joint QTL
M22
BY
RM
YPS163
YJM145
CLIB413
YJM978
YJM454
YPS1009
I14
Y10
PW5
273614N
YJM981
CBS2888
CLIB219
chrI
chrIII
chrV
chrVII
chrVIII
chrX
chrXI
chrXIII
chrXIV
chrXVI
genomic position

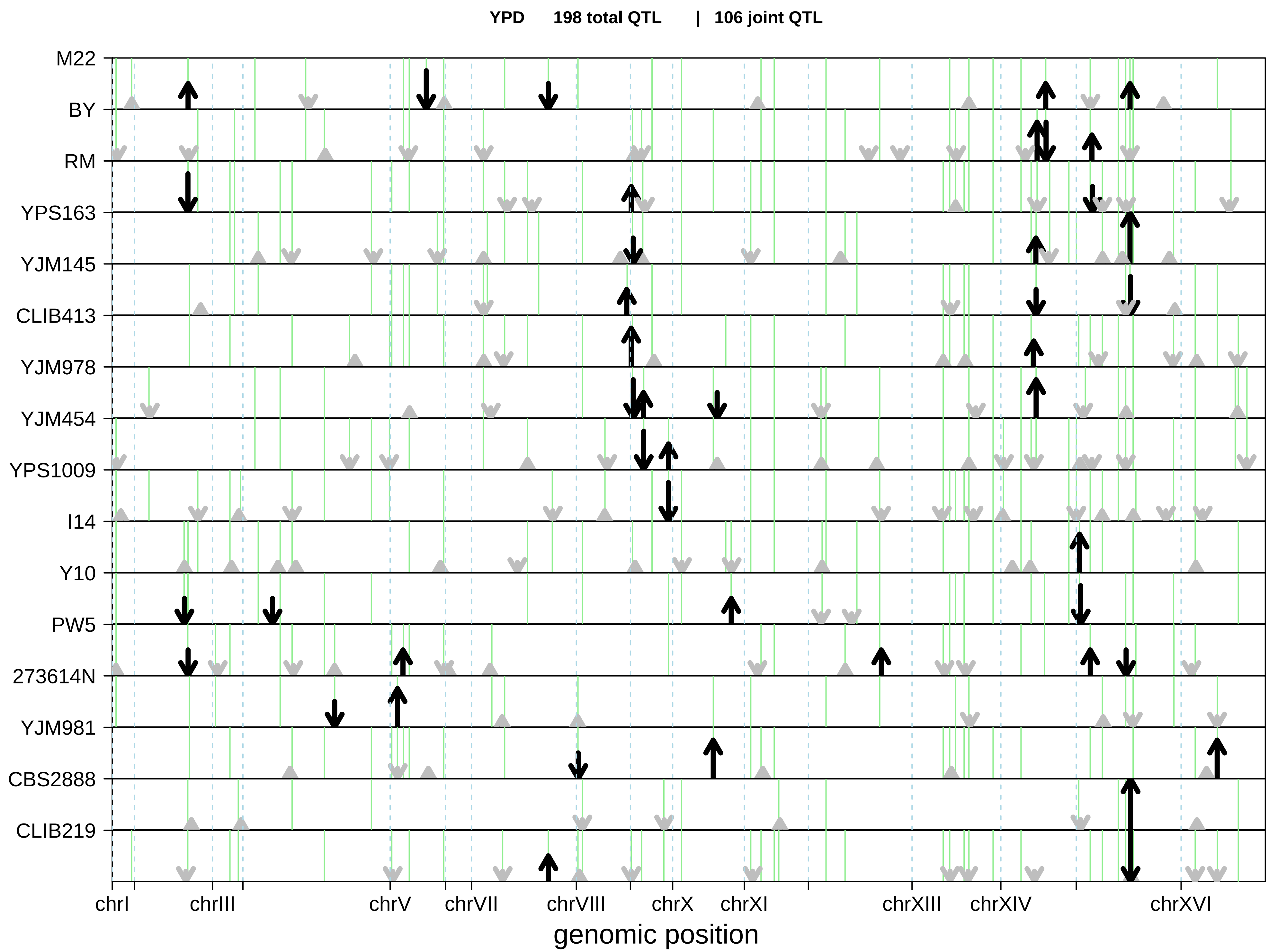

YPD 198 total QTL | 106 joint QTL
M22
BY
RM
YPS163
YJM145
CLIB413
YJM978
YJM454
YPS1009
I14
Y10
PW5
273614N
YJM981
CBS2888
CLIB219
chrI
chrIII
chrV
chrVII
chrVIII
chrX
chrXI
chrXIII
chrXIV
chrXVI
genomic position

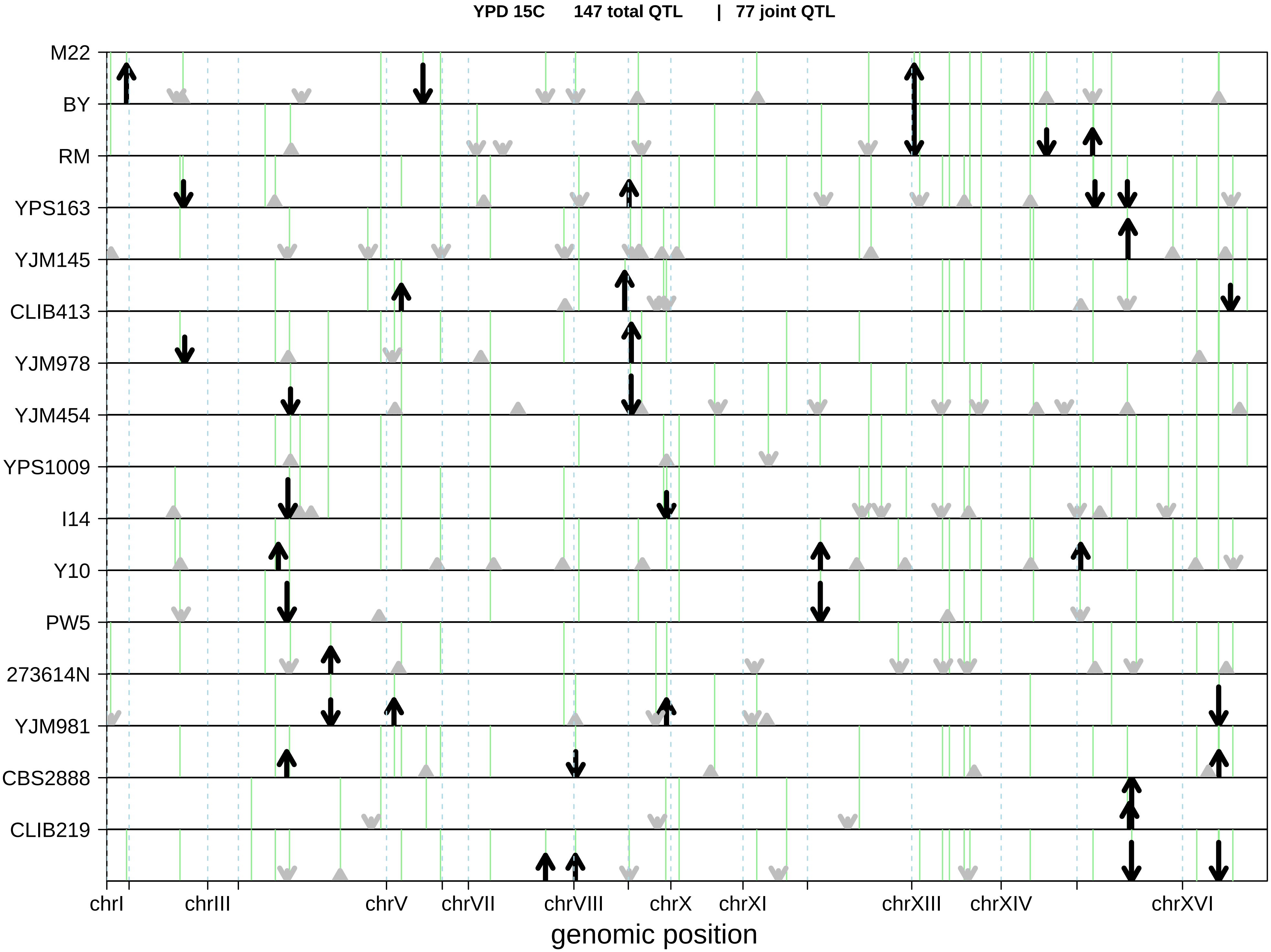

YPD 15C 147 total QTL | 77 joint QTL
M22
BY
RM
YPS163
YJM145
CLIB413
YJM978
YJM454
YPS1009
I14
Y10
PW5
273614N
YJM981
CBS2888
CLIB219
chrI
chrIII
chrV
chrVII
chrVIII
chrX
chrXI
chrXIII
chrXIV
chrXVI
genomic position

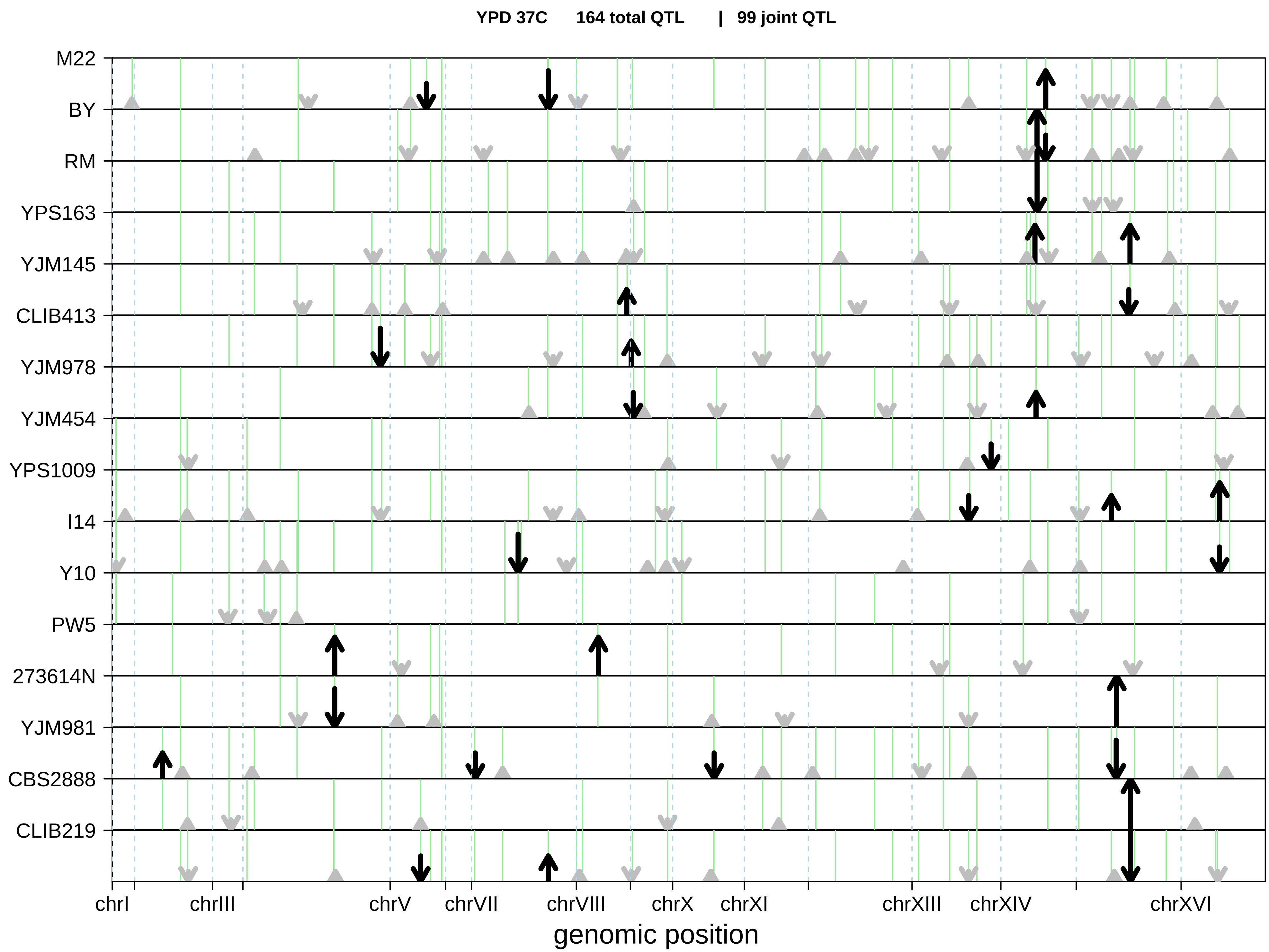
YPD 37C 164 total QTL | 99 joint QTL
M22
BY
RM
YPS163
YJM145
CLIB413
YJM978
YJM454
YPS1009
I14
Y10
PW5
273614N
YJM981
CBS2888
CLIB219
chrI
chrIII
chrV
chrVII
chrVIII
chrX
chrXI
chrXIII
chrXIV
chrXVI
genomic position

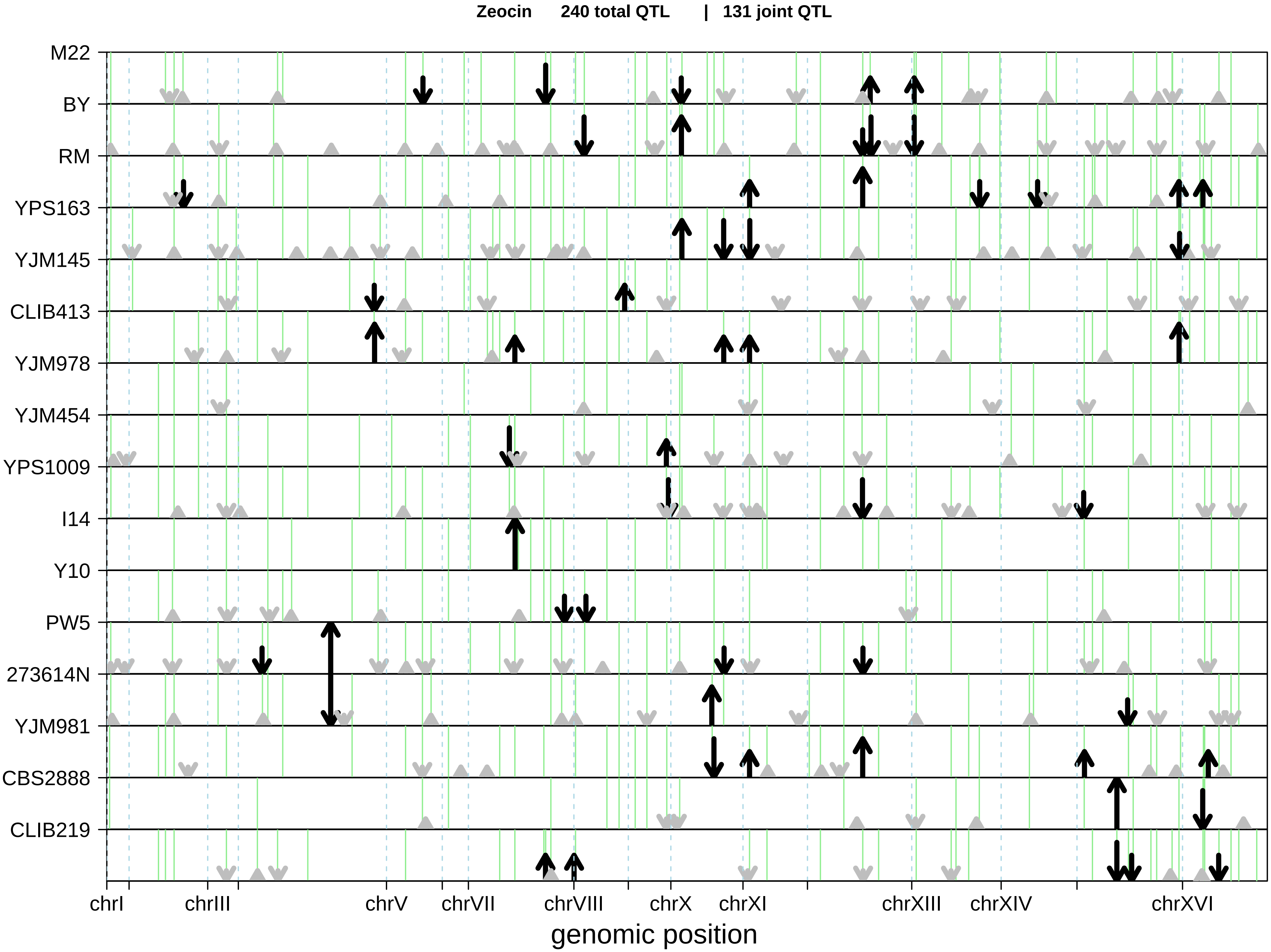

Zeocin 240 total QTL | 131 joint QTL
M22
BY
RM
YPS163
YJM145
CLIB413
YJM978
YJM454
YPS1009
I14
Y10
PW5
273614N
YJM981
CBS2888
CLIB219
chrI
chrIII
chrV
chrVII
chrVIII
chrX
chrXI
chrXIII
chrXIV
chrXVI
genomic position
